# A realist evaluation of community champion and participatory action approaches during the COVID-19 pandemic

**DOI:** 10.3389/fpubh.2024.1355944

**Published:** 2024-06-13

**Authors:** Neil Howlett, Olujoke Fakoya, Charis Bontoft, Isobel Simmons, Lisa Miners, Adam P. Wagner, Katherine Brown

**Affiliations:** ^1^Department of Psychology, Sport, and Geography, University of Hertfordshire, Hatfield, United Kingdom; ^2^Norwich Medical School, University of East Anglia, Norwich, United Kingdom

**Keywords:** community champions, realist evaluation, COVID-19, community participatory action research, COVID-19 champion, vaccine champion

## Abstract

**Background:**

During the COVID-19 pandemic, public health teams tried several approaches to circulate accurate health information and engage with community members to understand what they need from public health services. Two such approaches were community champions and community participatory action research (CPAR). This study evaluates two champion programmes and a CPAR programme in terms of what worked, for whom, and in what contexts, including the funding and resourcing associated with implementation.

**Methods:**

Between June 2022 and June 2023, a realist evaluation of three distinct case studies (COVID-19 champions, Vaccine Champions, and CPAR programmes) in the city of Southampton in England was conducted in three stages: development of initial programme theories and collection of additional contextual information, including funding and resources associated with delivering each programme; initial programme theory testing; synthesis of final programme theories. Data was collected primarily through semi-structured interviews (*n* = 29) across programme and training leads, voluntary services, community organisations, volunteers, and local community members, and one focus group with local community members (*n* = 8).

**Results:**

The City Council used £642 k from two funding awards to deliver the programmes: COVID-19 Champions £41 k; Vaccine Champions £485 k; and CPAR programmes £115 k. Twenty-eight initial programme theories were generated, which were “tested” to support, refine, or refute context-mechanism-outcome relationships, resulting finally in a set of 22 programme theories across the three programmes. Six demi-regularities were generated, each featuring in multiple programme theories, and providing data on how and why these programmes can work, and in which contexts: (1) building trust through community connections; (2) fostering relationships and collaboration; (3) provision of training and resources; (4) local community knowledge and expertise; (5) community representation and leadership; (6) appropriate communication and information sharing.

**Conclusion:**

This study provides new knowledge and understanding of the factors affecting the implementation of community champion and CPAR approaches during public health emergencies. These findings suggest that representation and involvement of community members, establishing and building on trust, adequate training and resources, and clear communication from trusted community members and organisations are catalysts for meaningful engagement with communities.

**Evaluation registration:** Research Registry identifier: researchregistry8094.

## Introduction

By March 2020, community transmission of COVID-19 in the UK, had become so widespread that unprecedented measures to control the spread and reduce pressure on the National Health Service were implemented. A series of lockdowns began on 23rd March 2020 and lasted until the summer of 2021. Throughout this period and beyond, local authority-based Public Health departments in England were responsible for co-ordinated COVID-19 responses, and a range of local initiatives and actions were implemented as part of “Contain Outbreak Management” plans, informed by knowledge about local population groups that might be most vulnerable and their needs. Two examples of these initiatives were community champion approaches [see (1), for an overview of COVID-19 champion programmes across London] and Community Participatory Action Research (CPAR) approaches, which train community members as researchers to explore topics of interest with their fellow community members.

### Community champion programmes

A variety of community champion programmes have been applied in the UK and internationally, during acute emergencies and for prevention on a broader timescale (2). These roles have been traditionally referred to as community health workers, who are individuals from the community in which they are working, are not professionally trained, and are normally volunteers (3). Embedding community health workers into community and health systems, and providing adequate supervision and training, can facilitate improved programme delivery in lower-to-middle income countries, however, information on how best to adopt such approaches is lacking (3). Agarwal et al. (4) proposed a conceptual framework for measuring performance of community health workers, focused on processes such as community health worker development and support from community groups, and outputs such as community health workers competency and community access, that can help improve community health outcomes.

More recently, these roles have been called champions or community champions. Public Health England categorised champion programmes into two broad approaches (2). The first is the “Popular Opinion Leader” approach, which utilises well-connected leaders who are already established in the community and play a role in health promotion. This model was most often adopted by Vaccine Champion programmes during the pandemic. This approach utilises staff and community leaders in a range of healthcare, third sector, or faith-based contexts. The activities delivered on these programmes are often structured information provision sessions [see (5) for an example]. The second is the “Community Mobilizer approach”, which utilises a wide range of volunteers, typically to support prevention and outreach, and allows reciprocal information sharing between communities and stakeholder organisations [see (6) for an example]. This model was most often adopted by COVID-19 champions programmes, which tend to utilise a large number of volunteers or lay-workers, to facilitate broad reach into communities, but with less structured activities. Both models can reach and communicate with target communities, through greater social connections and better linking of communities and services (2). However, the two models operate with different methods.

COVID-19 champions are able to reach target groups, communicate health risks, and understand and deliver solutions that are appropriate to their communities (7). These programmes are more likely to succeed when trust of the government is low, and champions are given autonomy and are seen as trusted sources (6, 7). A synthesis of practice-based learning in COVID-19 champion approaches found that stronger relationships with communities was a catalyst for prevention efforts in under-served groups, and that this can be facilitated by trusted community member involvement (8). Newham Borough Council in London were an early adopter of the COVID-19 Champion model and identified useful insights from champions, into why people got involved, how they communicated messaging onwards into the community, and unexpected benefits (9). However, there were challenges faced by COVID-19 champion approaches including: reliance on volunteers, potentially resulting in stress and burnout for them; limited resources; and reaching and including seldom heard and underserved groups (7).

Vaccine Champion programmes were a feature of public health efforts globally during the pandemic, and evaluations have focused on both the training of champions and the perceptions of service users. In Australia, vaccine champions, who were primarily healthcare and government workers, were trained to improve their knowledge and communication skills to become vaccine advocates (5). Following training, they felt more confident to discuss vaccine safety and effectiveness and to seek out additional information to fulfil their role (5). An example Vaccine Champion programme from India, was co-designed with community representatives to increase vaccine acceptance, and showed perceived benefits for both parents and caregivers, and champions (10). Parents improved their knowledge of the vaccine purpose and side effects and were more willing to travel for family vaccinations. Vaccine champions felt more ownership and were more able to tackle the concerns of community members (10). A more focused Vaccine Champion programme in Southeast London, was delivered solely through community pharmacies, and over four months engaged in several thousand vaccine-related conversations with community members, with the majority indicating willingness to have the vaccine (11).

### Community participatory action research programmes

An alternative approach to Champions to achieve better understanding of and connections to communities is CPAR, sometimes referred to as community-based participatory research or participatory action research. Ortiz et al. (12) highlight a conceptual model of community-based participatory research involving four domains: research context (e.g., capacity and readiness); partnership processes (e.g., relationships, partnership structures); intervention and research design consequent to shared decision-making (e.g., community-involved research); intermediate and long-term outcomes (e.g., shared power relations in research). It has also been recommended that participatory action research programmes are considered in three phases, involving design (involvement of stakeholder groups), implementation (stakeholders to focus on appropriate health impact and outcomes), and evaluation (participant perspectives and plans for sustainability) (13).

A review of community participatory approaches in health systems concluded that studies consistently highlighted improvements in the availability, accessibility and acceptability of services, with limited evidence for improvements in health behaviors or outcomes (14). In line with findings for community champion models, individual motivations, trust at the community level, and supportive institutional processes promoted community participation, while challenges highlighted included limited training, interest or information, and a lack of sustainable resources (14). In a later scoping review, a range of success factors were highlighted, which span across characteristics of partners (e.g., representation), relationships between partners (e.g., openness and transparency), and processes, resources, and outcomes of the partnership (e.g., sustainable community benefits) (15).

In terms of the research process itself, challenges can include a lack of time and financial resource to enable sustainable community engagement, and differing expectations, roles, and processes involved in partner organisations (16). Despite these challenges, there were factors that promoted community-based research partnerships, including recognition of stakeholder expertise, reimbursement of costs, and providing variety in communication channels and methods (16). When looking at the effects and processes involved in CPAR approaches, it is also important to explore benefits/outcomes at multiple levels, such as volunteers/paid researchers and community organisations. Volunteer researchers reported that their involvement in CPAR programmes was valuable training for community engagement and for experience in their health field of interest (e.g., future nursing related careers) (17). Community partners reported that utilising volunteers from within their communities helped understanding and acceptance of research-based approaches (17).

### Realist evaluation

Evaluating complex public health programmes, where assigning causation and isolating specific effects by controlling variables is not possible, necessitates a different approach than traditional randomised controlled trial design. Recent recommendations on rethinking the concept of evidence in implementation science suggest that evaluations should prioritise evidence that has been identified as important by the communities themselves, and the context of the programmes being evaluated as a key domain of study (18). Realist evaluations focus on “what works, how, in which conditions, and for whom” using context-mechanism-outcome configurations rather than focusing on outcome effectiveness alone (19, p.1). Contexts refer to anything that forms the existing backdrop of the programme or intervention and includes factors such as legal and political contexts, socio-demographics of those affected and social or cultural norms. Mechanisms combine the “reasoning” or reaction to “resources” inherent in the programme (19). Outcomes can include both intended or unintended consequences of the interaction between contexts and mechanisms. The three phases of a realist evaluation include: identifying initial programme theories in terms of context-mechanism-outcomes; testing the initial programme theories via data collection involving interviews with key stakeholders; and analysis of the context-mechanism-outcomes and building more refined programme theories (20).

Realist evaluations of community-based participatory research approaches in the context of health research and practice have previously been carried out. Jagosh et al. (21) reviewed studies on the benefits of participatory research and highlighted a middle-range theory (a synthesis across cases, the final phase highlighted in the previous paragraph) that focused on partnership synergy as the key catalyst for effective links between the process and outcomes of these approaches. Using this lens, findings indicated that participatory research can produce culturally appropriate research, increase capacity and competence in stakeholders, improve outputs and outcomes, and promote sustainability of project goals (21). In follow-up work, Jagosh et al. (22) showed that sustainability in community-based participatory research partnerships helped achieve collaborative efforts toward health improvement, spin-off projects, and system transformations at a population level. However, to the present authors’ knowledge this study represents the first attempt at a realist evaluation of community champion approaches, and of a CPAR programme within a pandemic context.

### Current study

Existing health inequalities have been further exacerbated by the COVID-19 pandemic and efforts to address these inequalities need to be sustained over the long-term and in partnership with those affected. Existing evidence about community-focussed initiatives such as Champion and CPAR programmes suggest that they can be effective for helping to improve reach and engagement with communities and could contribute to building more effective and acceptable services. Such initiatives, however, are complex and are introduced within dynamic contexts making evaluation of their effects difficult. To date, whilst there have been evaluations of wider community health champions and CPAR projects, published evaluations of their application in the COVID-19 context have been limited, particularly taking into account the delivery of multiple overlapping programmes in the same context. This study provides a unique contribution to knowledge by using a realist approach, alongside collecting information on funding and resources, to evaluate three co-occurring Champion and CPAR programmes in the city of Southampton in England, in the context of COVID-19. By using realist evaluation, this project aimed to provide unique insights that explain the context, mechanisms, and outcomes of such approaches, and draw out overarching themes across programmes, that can help to optimise service delivery in the future, informing both public health and academic research stakeholders.

The aims of the realist evaluation were to: (1) evaluate the COVID-19 Champion, Vaccine Champion, and CPAR programmes in terms of how and why they work, for whom and in what contexts, including information on funding and resources; (2) provide overarching themes that are present within and across these programmes to inform future service design.

## Materials and methods

This evaluation was funded by the National Institute for Health and Care Research (NIHR) Public Health Intervention Responsive Studies Team (PHIRST) programme, whereby local authorities apply to have one or more programme or service evaluated by a group of experts in public health evaluations.

### The three programmes

#### COVID-19 champions

In the city of Southampton in England, three programmes were adopted as part of efforts to tackle COVID-19: (i) COVID-19 Champions; (ii) Vaccine Champions; and (iii) the CPAR programme. The COVID-19 Champions initiative began in September 2020 and involved volunteers signing up to act as conduits into their communities for local information about the pandemic, current data on infection rates, and how people could protect themselves and those around them. It was also intended to be a way in which the local authority could get feedback from communities on the specific challenges they were facing, so that the local authority could form a better response to local need. Anyone was able to volunteer, and the local authority adopted a universal approach, without targeting specific groups or communities, recruiting through an online sign-up page on their website. All those signing up received details of a champion Facebook page and WhatsApp list. The WhatsApp group was partly utilised as a way of sharing the latest images of communication campaigns from local and national health agencies. They also received a weekly email bulletin which contained the latest data on COVID-19 rates in Southampton and England, reminders of the current restrictions, and information about briefings and online groups available that week. COVID-19 champions did not receive any incentives or reimbursement. By the end of 2022, the programme had evolved into a broader health and wellbeing champion model.

#### Vaccine champions

Vaccination to protect people from COVID-19 began in the UK in December 2020 (23), with an intensive two-dose programme roll-out that lasted into summer 2021. A booster dose roll-out followed in late 2021 into early 2022. Booster vaccinations for older adults and those with underlying health conditions will likely continue, for the foreseeable future. Although the UK achieved high levels of vaccination, certain groups were less likely to be vaccinated (for example people from Black and other minority ethnic groups) (24). The Vaccine Champion programme began in February 2022 (and overlapped the COVID-19 Champion programme). The purpose of this initiative was to increase COVID-19 vaccination rates, particularly amongst communities and groups where vaccination uptake had been lower. The approach was proactive, with deliberate efforts to engage organisations linked to communities with large numbers of unvaccinated people. This entailed a two-tier champion approach with a group of champion organisations grant funded for their time in order to reach and engage community members, with a second tier of unfunded businesses and organisations helping to distribute leaflets.

Once signed up, grant-funded champions were given details of a Facebook page and WhatsApp list and received a weekly bulletin by email containing champion stories, and information about vaccination hubs, vaccine pop-up sessions, and walk in clinics. Champions were also provided with weekly flyers which signposted residents towards local vaccination sites, communication in a variety of formats (e.g., social media posts, digital campaigns) and translations, Making Every Contact Count training, access to training on opening up conversations about vaccinations, informational videos, podcasts, weekly drop-in sessions and a dedicated email address to answer queries, and local data and intelligence. Subsequently, these organisations delivered a variety of activities such as sharing information about local vaccination provision, information sessions to dispel vaccine myths, tailored support to address cultural barriers to vaccinations, and even extended opening hours for vaccine provision in areas of low uptake. Some Vaccine Champion organisations provided educational and health and wellbeing activities, where conversations about vaccination could occur but not be the focus. For example, a sporting foundation provided tutoring for Maths and English. Funding for the Vaccine Champions work ran until March 2023.

#### Community participatory action research programme

The CPAR programme was funded from February 2022 to June 2022. It involved commissioning of a national not-for-profit community research and social innovation organisation to recruit and train peer researchers from local communities. Peer researcher training was facilitated and designed by the training organisation, with support by the City Council. Training was delivered over six weeks, with 8 h of training per week divided evenly between live zoom sessions and self-study/tasks. Training was supplemented by an extensive peer researcher handbook, which focused on the following topics: what peer research is; recruitment of participants; introducing themselves; gaining consent; safeguarding; interviewing skills; fieldwork checklists; participant information sheets; reflection log; peer research principles; and resources and further reading. The City Council also provided three sessions on: COVID-19, health inequality, and deprivation in Southampton; “what is public health”; and wider determinants of health. The peer researchers were mentored through the work by the community research organisation that recruited them. Five local voluntary sector organisations were involved to help reach, recruit, and engage peer researchers. The purpose of the community research was to understand five pre-determined issues affecting health and wellbeing during the pandemic (e.g., intergenerational experiences, digital use/literacy) so that local public health services could be tailored more effectively going forward.

### Study design overview

There are three broad phases involved in a realist evaluation (20). The first phase seeks to identify and formalise a set of initial programme theories. Data is gathered from individuals involved in the development of the programme, its key stakeholders, and from academic literature. This data is then used to build initial programme theories about the causal relationships between different contexts, mechanisms, and outcomes. These theories are then “tested” in the second phase through realist interviews with stakeholders to determine how the programme unfolds in real life contexts. In the third phase, the programme theories are refined through analysis and interpretation of the data to generate specific patterns known as demi-regularities (or themes), which are then linked to relevant research and theory.

#### Phase 1: Development of the initial programme theories and collection of costing and resource information

Development of the initial programme theories was completed through complementary approaches including: (1) review of documents related to the Champion and CPAR programmes that describe the programme rationales and protocols; (2) informal discussions with key stakeholders, e.g., programme managers, to identify assumptions about how the programmes work and in what contexts, to achieve their intended outcomes; and (3) consulting relevant published research.

##### Funding and resourcing overview of programmes

Information on funding and resourcing was collected for each programme, alongside any available demographic data. For the three programmes, information was collected from the relevant programme managers (outside of interviews), focusing on their provided budgets and what was delivered using them. Data from programme managers provided context, budget, and general service information, with follow-up contacts to sense check findings.

#### Phase 2: Testing and refining of initial programme theories

Realist interviews were conducted with key stakeholders including staff involved in setting up and delivering the programmes, COVID-19 champions, individuals representing Vaccine champion and CPAR organisations, peer researchers, and community members to provide a local perspective. Realist interviews started with exploratory questions to try to ascertain how the programme works for whom and in what circumstances. As the interviews progressed, the questions were more tailored to specific context-mechanism-outcome configurations from the initial programme theories most relevant to the interviewee’s experience.

Respondents were selected for the perspective and insight they may have about how and why the programme may (or not) work (25). It was important to obtain the perspective of different stakeholder groups as a variety of perspectives are needed to investigate informal patterns and unintended outcomes (25). Practitioners (e.g., public health team, community organisation staff) were seen as having specific ideas on what was within the programmes that works (mechanisms) as they were more likely to have a broad experience of successes and failures. Frontline practitioners (e.g., COVID-19 Champions, Vaccine Champions, CPAR researchers) were also more likely to be good sources of information about the programme barriers and unintended consequences (25). Local community members were more likely to be sensitised about possible outcomes and how programme mechanisms may have influenced outcomes (20).

A realist interview schedule was developed based on the template from the Realist And Meta-narrative Evidence Syntheses II Project (26). The realist schedule was tailored to each stakeholder group (e.g., programme managers, champions). Whilst realist interviews are qualitative in nature, their purpose is different to other types of interviews (e.g., constructivist) where the aim is to elicit and understand the respondent’s world view and experiences (26). Realist interviews investigate propositions about how, where, when, and why (not) programmes are successful, by capturing the participants’ stories about the programme (26). To do this, the interviewer relates with interviewees in a distinctive process called the “learner-teacher” cycle (20). The interviews explored stakeholders’ accounts of the programmes, their implementation, how the programmes were expected to work, barriers and its anticipated impact on practice. Interviews and focus groups were conducted either face-to-face and recorded via a Dictaphone or virtually and recorded via Microsoft Teams or Zoom. Transcripts were produced verbatim and pseudonymised at the earliest opportunity to remove identifiable information.

#### Phase 3: Programme theory specification

Data previously collected in phase 2 were analysed and integrated to develop a comprehensive understanding of the refined programme theories. An extensive examination of the programme theories was carried out to identify recurring patterns and similarities within the refined initial programme theories. Subsequently, within the grouped programme theories, specific patterns known as demi-regularities (or themes) were identified. The resulting demi-regularities were used to identify relevant background research and theory that reported on related causal chains or moderating factors which Marchal et al. (27) described as a “plausibility check”.

### Data analysis

#### Realist analysis

The audio from recordings of interviews and focus groups were transcribed by a General Data Protection Regulation compliant transcription service to produce verbatim transcripts. Adhering to realist methodology (25), the data analysis process utilised a retroductive approach, supported by both inductive and deductive analytical processes with the interview transcripts, while also incorporating the researchers’ own understanding to uncover generative causation. The process required researchers to move back and forth between the initial programme theories and the data, to identify elements of contexts and mechanisms that explain the outcomes (28). The analysis and synthesis of data followed guidance by Gilmore et al. (29), which allows for a transparent and rigorous analysis process to be conducted. NVivo, a qualitative data analysis software, was used for coding the interview transcripts. Coding of transcripts was completed in two ways. Excerpts relevant for initial programme theories were coded to the relevant programme theory (deductive), and then any other comment that was assessed as being a potentially useful additional context, mechanism, or outcome was coded (inductive).

The following step-by-step process was followed for each initial programme theory:

1. A “node” was created for each initial programme theory (identified in phase 1).
2. Any relevant comment related to an initial programme theory was coded to the relevant node.
3. All data attached to each node representing an initial programme theory was reviewed and if clear context-mechanism-outcome components could be extracted from the data, a memo was created and linked to the relevant initial programme theory to record the rationale for agreement, refinement, or refutation of the theory.
4. Memos for each initial programme theory were reviewed by a second member of the research team to ascertain whether each initial programme theory needed refinement, taking forward in its current form, removing where there was no evidence to support, or where there was overlap with another initial programme theory and could be combined.
5. A refined list of programme theories was produced by one member of the research team, and then independently reviewed by another. Any outstanding issues were discussed in a larger team meeting to reach consensus.

Recurring patterns and similarities within the refined list of programme theories were then used to produce overarching demi-regularities, with data from multiple programme theories (and stakeholders) supporting. Once demi-regularities were identified, previous research and theory were searched to enhance the explanatory power of the study, and to transition the evaluation from case-specific programme theories to middle-range theories.

#### Analysis of funding and resource data

Information on funding and resourcing provided by programme managers was summarised for a high-level overview. Where needed, other quantitative data (e.g., volunteer champion demographics) is summarised using descriptive statistics.

### Patient and public involvement and engagement

Patient and public involvement and engagement was embedded through both the involvement of three members of the PHIRST Connect Public Involvement in Research group and wider lay and public contributors recruited to a project specific “Public Voice” group, and a later dissemination and feedback event. Two members of the Public Involvement in Research group supported project development since inception, attending project meetings, commenting on the protocol, and one member (IS) was involved in developing the evaluation approach, interview schedules, supporting data analysis, and co-authoring this manuscript. For the public voice group, eight people were initially recruited, with a mixture of local residents, champions, and peer researchers. The group advised on the early development, delivery, and recruitment of other public members for the evaluation. The chair of the Public Involvement in Research group led the public voice group and attended advisory and project steering groups.

## Results

### Phase 1: Development of the initial programme theories and collection of costing and resource information

Between June and September 2022, an initial set of 28 programme theories (see Supplementary Table S1 for full list) was generated based on data gathered from individuals involved in the development and running of the programmes, other key stakeholders, and from academic literature.

#### Funding and resourcing

Southampton City Council used £157 K from the Department of Health and Social Care’s award of the “Contain Outbreak Management Fund” (2020–2021 and 2021–2022) and the Department for Levelling up, Housing and Communities award of £485 k “Community Vaccine Champions Scheme” funding (2022) to deliver the three community approaches.

**Figure 1:**
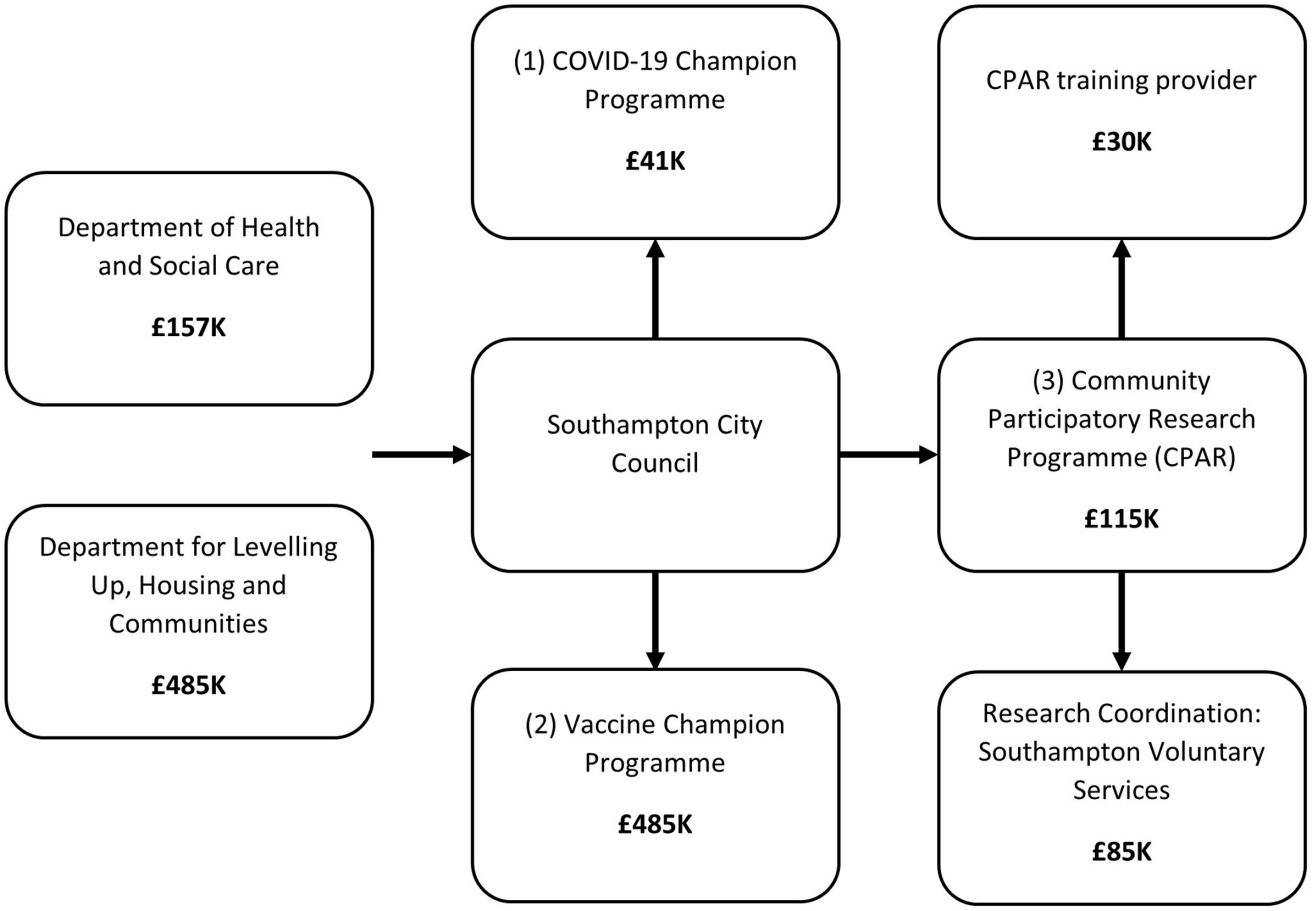
The application of funds across the three champion/CPAR programmes.

Figure 1 depicts the funding division for each programme based on information from project leads at the City Council.

##### COVID-19 champions

The City Council was awarded £41,725 to support delivery of the COVID-19 champion programme. This total amount was used to pay the salary (including oncosts) of a community engagement officer who was involved in administrating and coordinating the programme and its champion volunteers. As of February 2021, there had been 306 COVID-19 champions signed up to participate. Limited demographic data on champion characteristics was collected by the City Council. English was the predominantly spoken language (87%, 265/304) followed by Polish (3%, 8/304) and Spanish (2%, 7/304), but there was representation from 26 different languages. More detailed data was available on a smaller subset (*n* = 208) of champions who opted to share their details, whose median age was 55–59 years. Their reported ethnicity was: 88% (183/208) White; 5% (10/208) as Asian; 3% (6/208) as Black African/Caribbean/Black British; 1% (3/208) as Mixed and the remainder as “other” (2%; 5/208).

#### Vaccine champion programme

The City Council allocated £485 k to the Vaccine Champion programme, which funded time for 12 staff members to recruit local vaccine organisations, organise grant allocation and support programme delivery (e.g., a public health consultant, project manager, and two project support officers). In total, £226 k was awarded in grants to 23 local organisations, with mean and median values of £9.8 k and £9.3 k, ranging from £300–£53,140. These organisations were often embedded in underserved community groups (e.g., a mosque, two organisations supporting refugees, an Afro-Caribbean Women’s group). This funding paid for a variety of outreach work that aimed to embed discussions about vaccinations into routine activities and communications, with larger funding allocations also being utilised for additional events centred around encouragement for vaccination and wider talks about health and wellbeing. Additionally, there were 105 local businesses who, with no reimbursement, agreed to share the “One Southampton” communications campaign in the form of leaflet distribution across the city.

#### CPAR programme

The City Council allocated £115 k of funding to the CPAR programme, with local Voluntary Services awarded £85 k for coordination (and reimbursing peer researchers for their time) and a national community research and social innovation organisation awarded £30 k to deliver training and mentoring of peer researchers. There were three broad phases to the CPAR programme: recruitment; training; and conducting research. During recruitment, the training organisation worked with the five local community organisations to recruit peer researchers. Together, they created and shared adverts, asked potential peer researchers to complete application forms and carried out interviews with community organisations. It was estimated that across a month, three members of the training organisation worked a total of 10 days to complete recruitment (5 days of which focused on interviewing potential peer researchers).

Peer researcher training was facilitated and designed by the training organisation, with support by the City Council. Training focused on the topic, research skills, ethics and conducting research. Training was delivered over six weeks, with each week consisting of four one-hour zoom sessions and around four hours of self-study/tasks. Interviews and focus groups for the research were completed by 15 peer researchers over a three-month period. Peer researchers were affiliated and mentored by community organisations, with further “drop-in time” provided by the training organisation. The decentralised management of peer researchers meant that the total numbers of interviews were not recorded. Those recruited typically worked around 2–3 days per week for up to 3 months and were paid the current UK living wage^1^ for their work.

### Phase 2: Test of initial programme theories

A total of 29 one-to-one interviews and one focus group, with 8 local community members, were conducted between September 2022 and April 2023. This covered 20 coordinators and 17 community members. The breakdown of coordinator roles, and overall coordinator and community member demographics are provided in Table 1. Given the age and health status of the community members, this sample can usefully inform on the perspective of older ages and long-term conditions, known risk factors for COVID-19.

**Table 1:** Interviewee participant demographics (*N* = 37).

Stakeholder group	Stakeholder subgroups	Age (years): mean (range)	Sex: *n* female (% female)	Ethnicity: *n* (%)	Long-term condition or disability: *n* (%)
Coordinators (*n* = 20)	Programme leads (*n* = 3)	48 (22–70)	13 (65%)	White British, 10 (50%) White other 3 (15%) Asian 3 (15%)	n/a
	Training lead (*n* = 1)				
	Voluntary services lead (*n* =1)				
	Community engagement officer (*n* = 1)				
	Community organisation representative (*n* =2)				
	Vaccine champion (*n* = 3)				
	COVID-19 champion (*n* = 5)				
	Peer researcher (*n* = 4)				
Community members (*n* = 17)		72 (34–87)	9 (56%)	15 (88%) White British	11 (69%)

### Phase 3: Synthesis of programme theories

Across the three programmes a final total of 22 programme theories were generated (see Supplementary Table S2 for a detailed summary) between April and June 2023. From the initial 28, each initial programme theory was either: taken forward in its current form (2 instances); refined (20 instances); removed where there was insufficient evidence to support (4 instances); or combined with another initial programme theory in cases of considerable overlap (2 instances). To make the programme theories more accessible to project partners and wider audiences, a plain English summary and take-home messages were produced for each (Supplementary Table S2). This was shared with the evaluation steering and advisory groups so they could provide feedback and action any learning for current and future programmes. Synthesis of these programme theories across programmes produced six demi-regularities.

#### Demi-regularities

We present each of the six demi-regularities with a narrative summary, linked to existing research and theory, with illustrative quotes, providing interpretative depth. The quotes presented are the most representative of each demi-regularity. A summary of the six demi-regularities is provided in Table 2, linked to selected programme theories, with 10 out of the 22 programme theories presented as they are most relevant for each quote (see Supplementary Table S2 for full list).

**Table 2:** Demi-regularities summary and selected relevant context-mechanism-outcome configurations

Demi-regularity label	Demi-regularity summary	Programme theory (PT) and label	Context	Mechanism	Outcome
1: Building trust through community connections	Increased levels of trust can come from community organisations and individual members being embedded within existing community groups with which champion and peer researcher programmes were hoping to engage.	PT2: getting representative champions and peer researchers	If recruited champions/peer researchers are a good representation of targeted communities.	**Resource:** champions/peer researchers will have more commonalities with the community and will be more likely to have the confidence, knowledge, and understanding to approach the communities **Reaction:** this could lead to increased trust among members in the community who would perceive champions/peer researchers as more relatable.	This can lead to increased engagement from community members in the champion/peer researcher programme.
2: Fostering relationships and collaboration	The importance of working together was recognised, with community members and organisations trying to achieve better health outcomes and create sustainable change in the community.	PT18: partnership working improved	If appropriate support structures are in place for the CPAR programme to engage with local organisations.	**Resource:** the CPAR programme provides a tangible way for public health programmes to work closely with community organisations.	This can lead to effective collaboration and ongoing partnerships with community organisations.
		PT7b: insights must lead to improvement of services	If insights from community members about how best to deliver current programmes, and potential barriers are gained through champions/peer researchers.	**Resource:** there are no actions taken in terms of changes to the current programmes or future services. **Reaction:** community members feel disappointed by the lack of actions/improvements.	Uptake or engagement is negatively affected for current programmes and future services among target communities, groups, or individuals.
3: Provision of training and resources	Providing adequate resources and training (where appropriate), helps to ensure champions and peer researchers have the capacity, skills, and knowledge necessary to effectively communicate with community members and build relationships based on trust and mutual respect.	PT20b: resources and training for community research	If there is adequate resource for the CPAR approach.	**Resource:** effective training and ongoing support for community researchers. **Reaction:** CPAR researchers are able to conduct safe, ethical, confidential, and open-ended interviews.	The CPAR programme is viewed as trustworthy and community members feel comfortable being open and honest in interviews, providing valuable insights.
		PT4: enabling champions and peer researchers	If champions/peer researchers who might (or not) have prior relevant experience are adequately trained in the role.	**Resource:** timely training materials and resources are provided, with opportunities to practice and access to ongoing support or mentoring. **Reaction:** champions/peer researchers would feel more confident and enabled to deliver an effective service.	This could lead to more effective reach into communities and communications with community members.
4: Local community knowledge and expertise	Community member involvement is essential for success because they bring local knowledge and expertise that is crucial for delivering programmes that meet the unique needs of their communities.	PT6: two-way understanding and ownership	If there are representative community members engaging in the programmes.	**Resource:** this provides a forum for local authority and partner organisations and community members to listen to and understand each other, often through champions. **Reaction:** local authority and partner organisations would feel more confident understanding how to meet community member needs, and community members would feel more empowered and trusting of services.	This improves relationships between local authority and partner organisations and community members and brings about a sense of shared ownership of programmes/services.
5: Community representation and leadership	Community representation and leadership helps to build trust and credibility, as these individuals and organisations are able to communicate with the public in a way that is respectful and culturally appropriate.	PT16: representation through vaccine champion organisations	If vaccine champion organisations already represent local community members and/or issues of importance (e.g., health, faith).	**Resource:** they are ideally positioned as trusted sources to initiate conversation and circulate key information. **Reaction:** community members are more likely to pay attention to messages they hear from trusted sources, especially where direct contact has been made.	A trusted source is gained in the champions, who are actively involved in local matters. This can lead to community members making more informed decisions, thereby increasing engagement and impact.
		PT3a: embedded champions and peer researchers	If recruited champions/peer researchers are already well known in their communities/established leaders.	**Resource**: champions/peer researchers will have existing links and networks with people across the target community **Reaction:** community members will feel safe and be more receptive of champions/peer researchers as they would be perceived as trustworthy.	This could lead to increased uptake and meaningful reciprocal engagement between champions/peer researchers and community members, which can last over the long-term
6: Appropriate communication and information sharing	Communication and information that is both culturally appropriate and/or that utilises the right delivery formats is an important consideration to build and enhance trust and credibility with community members.	PT15: making informed choices	If information about the vaccine is not readily accessible to individuals in the community.	**Resource:** champions can act as the bridge in providing key information to individuals within the community and feedback the community voice. **Reaction:** individuals in the community would feel that they have better understanding and knowledge of the vaccine and related local and country wide issues.	This would likely lead to individuals being able to make a more informed choice about vaccination and the associated community benefits.
		PT12: personable and appropriate communication	If individuals in the community do not feel as though they have enough vaccination information, or the information keeps changing.	**Resource:** communication with champions and peer-researchers provide personable, accurate and up-to-date information without pressure, or signposting to a suitable health professional who can provide specific information about vaccine side-effects. **Reaction**: individuals trust the Champions and peer-researchers and feel well-informed.	This can lead to better understanding of the vaccine and stronger intentions to get the vaccine.

##### Demi-regularity 1: Building trust through community connections (selected programme theory: 2)

Being embedded within existing community groups can be due to an organisation already serving that community, or an individual being representative, well-known, or an established leader within a community. In this circumstance, the individual is able to build on relationships and trust that already exist within the community. Offering information from trusted sources is recommended as a strategy to increase vaccination uptake in hesitant community members (30). Moreover, by partnering with established community organisations, these programmes can leverage the trust that these organisations have already built with their members. Members of the community are more likely to trust and engage with a programme when it is led by someone they know and respect, or an organisation that has a history of serving the community.

Quote: “*Well, the first thing is really trust, isn't it? Because we have to trust each other to share the messages. So, the most problems with COVID would be overcome, I think a lot, not most, if we had that, because some communities, as we saw from CPAR research, when people go for information online, then problems start. So, it's sending them clear scientific data, and make sure that there is that trust. Obviously, you can't just barge into a community and say trust me because I look good. So, you need that relationship, which is built, and basically this is how Vaccine Champions really were created. This was part of it, like, how to engage with those communities, because they don't know us, they don't know the council. If anything, probably we're on the wrong side for them to understand us. So that's how these links with community organisations that are present in those communities, started creating*.” (Programme lead)

Quote: *“The stress that can come from going elsewhere or travelling elsewhere or not being in a place that feels safe to you, you're not going to trust what anyone says because you already feel like you're out of your comfort zone and you've got no power. So, enabling people to be sort of here, for example, or even in schools, so parents can come to a school and hear things about the vaccines for their children, they'll feel more able to trust.”* (Community member)

Increased trust was also highlighted in relation to champions and peer researchers being invested in the importance of the programmes and having personal experience of the health and wellbeing experiences or wider circumstances of the target community members/groups. This aligns with previous literature that has shown that when community partners are personally invested in the research, efforts to enhance trust become a priority, leading to the development of impactful working relationships (22). This can help to build trust and credibility with community members. When champions and peer researchers have shared experiences with individuals from the community, they may be more likely to be listened to and respected. Recognition of the impactful value of experiential knowledge from local communities has been found to be a successful indicator of sustainable community-based participatory research programmes (15).

##### Demi-regularity 2: Fostering relationships and collaboration (selected programme theories: 7b and 18)

Relationships and collaboration were key components of the Vaccine Champion, COVID-19 Champion and CPAR programmes. One way the programmes foster relationships and reciprocity is by linking established organisations and well-known individuals with communities. These organisations and individuals act as intermediaries, helping to build relationships and trust between the champions and researchers and the community. This is supported by a review of the UK evidence on community champions which found that they act as a link between communities and services and build social relationships (2). They provide insight into the needs and concerns of the community, and act as advocates for the programme. This can subsequently lead to effective programme implementation and better uptake by the community. In addition to this, these programmes provide a platform for collaboration between the local authority, community organisations, voluntary services and community members. Research suggests that the need to meet new demands during the early COVID-19 pandemic provided an opportunity for new partnerships and collaboration between organisations (31). This collaboration can potentially lead to better understanding of the needs of the community and the creation of more effective services.

Quote: “*The notion of handing power back to communities is one that really resonates with our mission, as a kind of anchor organisation within the voluntary sector. And I think what we've done is the public health team, the [name of organisation removed] and ourselves, we've created a real synergy there by all bringing our own organisational strengths to the programme. Along with, of course, the five supporting organisations and the peer researchers. So, I think it's a good demonstration of how together you can do things a lot better*.” (Voluntary Services lead)

It was also theorised that these programmes provided a platform or new forum to explore reciprocal working between the local authority, community organisations, voluntary services, and community members, and that this would lead to better services going forward. A meta-review of community participatory research identified partnership processes, including the importance of reciprocal relationships, as one of the key contributors to effective approaches (12). The idea of reciprocal working between different stakeholders is important for these programmes because it recognises that sustainable change cannot be achieved through a top-down approach. However, there is a danger that this relationship building will be wasted if community members and organisations do not see actions resulting from the collaboration (i.e., no change to services).

Quote: “*I think my biggest point that I want to stress, is what I was saying at the beginning about just the lack of feeling. It feels like the research has been quite inconsequential because we haven't seen anything being done about it. I haven't received any feedback on what I've done, and so it's hard to pass that back down to the community as well. So, I don't want it to be for nothing, and I'm sure that no one else in the project does, but that's what it feels like has happened, at the moment. So, potentially, some more work needs to be done there.*” (CPAR researcher)

##### Demi-regularity 3: Provision of training and resources (selected programme theories: 4 and 20b)

Studies reviewing how to optimise the use of community health workers support the notion that training, and ongoing support and supervision, which are suitably resourced, are essential to the success of these approaches (32, 33). Inadequate training or support structures, a lack of resources, and weak infrastructure have also been highlighted as barriers to community engagement for COVID-19 prevention and control (34). This provision also helps to create a sense of ownership and empowerment amongst the community members involved, which makes them more likely to take an active role in the programmes and to feel invested in their success. An appropriate level of training and ongoing support is particularly important in CPAR approaches so that research is conducted in a safe, ethical, and confidential manner. This helps ensure professional conduct, but also maximises the likelihood that community members will feel open to sharing their thoughts and feelings about the interview topic/s.

Quote: “*This isn't simply going out and anecdotally chatting to people, this was within a research, within a qualitative research framework. There was a lot of consideration that the peer researchers had to give to a lot of areas, not least of all ethics. So, if it's going to have validity as an exercise, it needs to be done properly, because otherwise there's so many ways that it could go wrong. People could be using unethical methods, people could not be getting the reach, the research questions might not be appropriate. I mean, the list could go on and on, couldn't it? So, it's really important that it has a solid foundation and that the peer researchers fully understand what their role is, that they're involved in it, because part of it is coming from them themselves. And they're interested in those communities because they're part of it, that it's safe for them and for the participants. And that the findings that are coming out of it are seen as valid and rigorous.*” (Programme Lead)

An additional consideration was that for the CPAR programme, the timeline was seen as very tight by involved stakeholders (i.e., the programme lead, and community organisation and researchers). The need for this type of approach to be resourced over a longer period for more sustained community engagement is an important learning point going forward. This is reflected in a report from the Scottish Government (31) that found organisations involved in the COVID-19 response want to prioritise “integrated and sustainable models” for services going forward. Early engagement (34) and the need to build trust over the longer term to achieve the best effects and sustainable partnerships (22) are both features of effective community participation efforts.

Quote: “*I think that this project that we worked on took place over a very short period of time. The training itself took four weeks, and then there were about three weeks left for community engagement. Unfortunately, the researchers were not encouraged to start contacting possible participants at the beginning of the project. So, this is something that I stressed that we should be doing—again, it didn't happen. So, we lost four weeks, and then we had three weeks to, frankly, scramble because we know it takes time for people [to engage with community members]. So, giving an adequate time to the project is essential. It took more time for the training and the wrapping up of the results, than it took for the actual community engagement and the research period. So, one third was actually searching the community, and two-thirds was training plus wrapping up, so disproportionate.*” (Community Organisation)

##### Demi-regularity 4: Local community knowledge and expertise (selected programme theory: 6)

The involvement of community members throughout the champion and CPAR programmes brought local knowledge and expertise that is crucial for delivering programmes that cater to community needs. This is supported by a review of volunteering during COVID-19 that found utilising local knowledge was one of the keys to identifying and responding to the needs of underserved groups (35). Through collaborative working, community members were able to share their experiences and insights, which helped shape ongoing development of programmes. This involvement helped to ensure that the programmes were culturally appropriate and sensitive to the needs of the community.

Quote: *“And in terms of the power sharing, the whole ethos behind the programme is that there's that shared power and shared decision-making. So not only are we pushing out messages, but we're listening really actively as well, so that we can then shift and change services. So, listening about which venues work well, listening about what kind of other health needs people might want to have met at those vaccination sessions, listening around what some of the barriers might be*…*I think it would. I think it would help make the programme more appropriately designed to engage more people across the communities, because there'd been that codesign early in the process.”* (Programme Lead)

Involving community members helps to create a sense of ownership and empowerment within the community. When community members are involved in programmes, they are more likely to feel invested in their success and take an active role in their implementation. Research on community-led responses to the COVID-19 crisis shows that local expertise can help reach vulnerable groups, direct help to where it is needed most, and extend the reach of traditional services (36). Consequently, a potential longer-term benefit, particularly from the CPAR programme, is that insights from the local expertise of the community members interviewed have the potential to improve future services and programmes, which would leave a legacy of community engagement in the city.

##### Demi-regularity 5: Community representation and leadership (selected programme theories: 3a and 16)

Community representation and leadership in champion and peer researcher programmes can help to overcome barriers to access and uptake of the programmes. This is supported by an evaluation of community-led responses to COVID-19, which showed that local leadership can be crucial in empowering action and coordination efforts through strong existing networks (36).

Quote: *“I mean because people relate to people that they think are like them don’t they? We all do that naturally, whether we recognise it or not, we do. We’re drawn to people with whom we feel relaxed I guess because they’re most similar to us and that might be for a whole variety of different things. It might be age, it might be geographical situation, it might be economic variants, it might be, you know, ethnicity, faith, all those things that make us feel comfortable and confident with particular people.”* (Vaccine Champion representative)

Quote: *“Then when I went to the mosque, they did say it’s good and you should get it done and, you know, everyone needs to get it done and they did give good advice, and then I just feel more confident about getting it done.”* (Community member)

Community leaders and representatives can provide insight into the specific challenges and concerns of the community, which can help to shape the development and implementation of current, and future programmes. Research on sustained community partnerships highlights the importance of shared leadership, and the perception that leaders are trustworthy and represent community interests (15). Community representation and leadership helped the champion and peer researcher programme goals align with the needs and values of the community. There were also instances in which community leaders and representatives served as role models, helping to build trust and promote vaccination uptake in the community.

Quote: *“I had my jab in front of them, and that was a good example. So, one of them, and no one was going up, so I went first and just did it because, for me, it's safe, it's good, and no one's trying to do anything… I believe it's right. So, when people trust you, some of them will come forward, and others will still be reserved and may not. But I think it's important for those champion leaders to be there, but in order to do that, you would have to have built up trust over years and years of that community. So, 14 years of running a breakfast and homeless, they trust you, they love you, so they know, and that's the way that you can, not entice them, and I wasn't saying they had to have it, or didn't have to have it, it's their choice, but it was safe to do it. And they know I'm not trying – there's nothing in it for me.”* (Vaccine champion representative)

Representation has been shown to be important both in previous research and from the data collected for this evaluation. The organisations involved in the Vaccine Champion and CPAR programmes represented leaders in their community, but the demographics of the COVID-19 champions, presented in the funding and resource section, suggest that recruitment of volunteers for this programme lacked diversity and may have struggled to fully represent the target groups.

##### Demi-regularity 6: Appropriate communication and information sharing (selected programme theories: 12 and 15)

Culturally appropriate communication ensures that everyone understands the benefits of vaccination and feels comfortable making an informed choice about getting vaccinated (or not). Research focused on addressing vaccine hesitancy recommends the use of culturally relevant information from trusted sources, available in multiple languages (30). However, it is important to acknowledge that even with optimal communication and access to information, not all individuals will be persuaded to get vaccinated. Hence, it is the responsibility of champions to foster an environment that facilitates informed decision-making, rather than attempting to change individual viewpoints about vaccination.

Quote: *“Well, yeah, I do think that's important, because otherwise how would people get that information? I mean, it's quite staggering, really, because we see people who are regularly in hospitals, they're regularly in GP surgeries, but still aren't necessarily aware of how and where they can get their vaccinations from. So, when they come into the centre, it's on a poster right in front of them that says, right, this week you can go to here, here and here, and you can just walk in when you're ready and get your vaccination, if you'd like to. And without that information here today, they may not be aware of it. So, there's lots of instances like that where we've been out and about at events, and we've made people aware that there's lots of different options.”* (COVID-19 Champion)

Effective communication helps to build trust and credibility with community members. Moreover, it can ensure that community members understand the purpose of the programmes, as well as the potential benefits to their health and wellbeing. By sharing accurate and up-to-date information, the programmes can help educate the community about the importance of vaccination and dispel myths or misinformation surrounding the COVID-19 vaccine.

Quote: *“I think conversations with Vaccine Champions for people that were unsure, I think there would definitely be a positive reaction. I think a lot of people would be persuaded to go and have their vaccine, or if not, certainly find out more about it and certainly understand why it's so important that people do undertake the vaccine programme, especially when you're looking at very vulnerable people, as we're dealing with within our organisation here, how important it is to protect them.”* (COVID Champion and Vaccine Champion representative)

The delivery format of communication and information sharing is also an important consideration. Although digital communication can increase reach, it may exclude large portions of particular target community groups. This is supported by research that suggests health inequalities can be worsened for groups that experience digital exclusion (e.g., older adults), who may have poorer health outcomes due to lack of access to digital healthcare and support (37).

Quote: *“Everything these days, not only just Covid but everything now, any information you get they’ll give you the web address and the web number or whatever and they don’t think of the, they think everybody’s got a computer. Well, the majority of the people where I live haven’t.”* (Community member)

**Figure 2:**
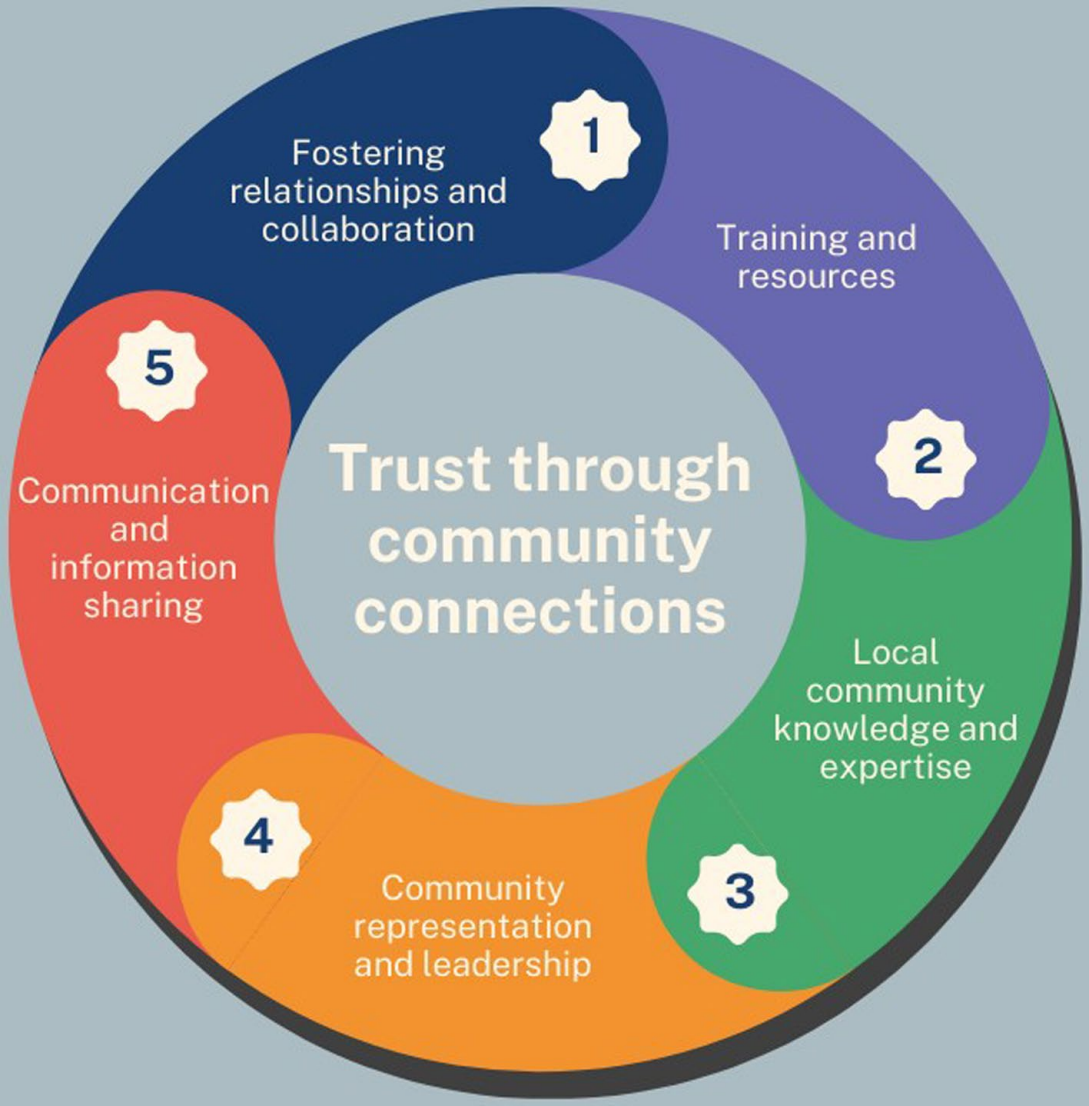
Building trust through community connections plays a key role in all other demi-regularities.

Having identified and developed the demi-regularities that reflect the core ways in which champions and participatory research programmes are able to bring about change within communities we reflected that the first demi-regularity, “building trust through community connections” represents a common thread that links all of the demi-regularities together. The process of initiating these programmes must start through engaging with existing community connections and building on this initial trust to achieve each of the other demi-regularities. As each of these is further developed it in turn supports strengthened trust and community connections, which is supported by recent findings from local and national COVID-19 champion programmes across England (6, 7). This is illustrated in Figure 2.

## Discussion

The City Council used two funding awards totalling £642 k to deliver the three programmes. The Vaccine Champion programme was much more resource intensive than the COVID-19 Champions requiring greater staff time, and utilising grants to maximise engagement with a range of local organisations. Most CPAR funding was allocated to paying peer researchers and their coordination by voluntary services. The six demi-regularities, found across cases and programme theories, showed the importance of *communicating* with people in a clear and open way. Relatedly, *sharing information* using emails or websites may help to increase reach overall, but may also mean that a lot of people will be excluded. The *local community knowledge and expertise* of people who live in communities is a rich source of information about what might be the best approaches to help, support, and engage underserved groups. *Fostering relationships and collaboration*, through community members working together with trusted local organisations, can lead to better understanding of the needs of the community and how to improve services. *Community representation and leadership* was seen as essential in community champion and peer research approaches because it is a catalyst for knowing about the challenges and concerns of the community members that they regularly talk to. *Provision of training and resources* for community members who signed up to be community champions or researchers, was crucial for them to feel empowered and capable in these roles. *Building trust through community connections* using community champion and research programmes is more likely when they are led by someone they know and respect, or an organisation that has a history of serving the community.

### Implications for policy and practice

As a pragmatic evaluation of real-world programmes that were implemented in particularly challenging circumstances this research provides several implications for those working in local authorities and voluntary services/organisations, and for those commissioning future services. Across all three programmes, the extent of the willingness to volunteer and the enthusiasm for engagement may have been partly driven by the unique pandemic context, and so future programmes may encounter additional challenges to recruitment and retention (e.g., less time available for volunteering; lower levels of motivation to help outside of an unprecedented crisis). In the case of the COVID-19 Champion programme, even the volunteers that did participate largely identified as White British. It may be that during public health emergencies those who are most likely to have capacity or resource will volunteer, and so volunteering is a reflection of existing health inequalities. Future programmes need to incorporate a variety of recruitment strategies to ensure more diverse volunteers, in terms of a wider range of ethnicity, age (i.e., younger volunteers), and other key demographics (e.g., faith or education). Consideration of payment for those from under-represented groups may be needed to promote more diverse champions going forward. Furthermore, although digital information sharing (largely used by COVID-19 champions) can increase reach, it is likely that large portions of the intended groups will be excluded if this is the only delivery medium. Although online communication can save short-term costs, it may also increase long-term health system costs by exacerbating health inequalities.

The Vaccine Champion programme cost around 12 times the resource of the COVID-19 Champion programme. A substantial proportion of funding was allocated to community organisations who put on structured activities such as pop-up events, walk-in clinics and wider education and health initiatives, representing a more intensive programme than the COVID-19 champion model. Provision of activities such as Maths and English tutoring, supporting low-income families with free food, and mental health support, may represent ways of reaching underserved groups that can be utilised in future champion programmes. Tentative evidence from the interviews suggested that through this greater funding and more intensive activities, better reach and outcomes may have been achieved (e.g., people being transported to vaccination centres or getting vaccinations at an event). It may be that this extra investment is cost-effective in the long run and that utilising key community organisations and leaders instead of volunteers may provide better representation of and penetration into underserved community groups. A recent report showed that Community COVID-19 Champion programmes may not impact vaccination rates (6).

There were key implications of appropriate resourcing, with data suggesting that future CPAR approaches should allow adequate time for meaningful engagement with and sufficient recruitment of community members. Furthermore, a plethora of research supports the notion that CPAR approaches work best over a long period of time, allowing ongoing engagement with the community, building research capacity, and exploring a range of issues [e.g., (14, 15, 22, 31)]. There is a real danger that if community members (and organisations) do not see any actions taken as a result of their community research, it will negatively affect future relationships and trust. There may also be a need to manage the expectations of the stakeholders involved, in regard to the extent to which change is possible for complex issues (e.g., housing). On a positive note, an accredited qualification offered by the training organisation was highly valued by the peer researchers who opted to complete this training and should be considered as part of an offer to community researchers in future CPAR approaches.

### Implications for research

This evaluation faced challenges in identifying participants who actually experienced the programmes directly. The programmes were not branded and advertised by name and so someone who may have been sent information on infection rates by a COVID-19 Champion, attended an event held by a Vaccine Champion organisation, or was interviewed for the CPAR programme, would not even know that they were a “service user”. The need to identify service users has to be balanced with the recommendation from Vaccine Champion evaluations that branding by the local authority should be avoided as it risks decreasing trust levels (38). Embedding more routine data collection of the demographics and reach of champions and community researchers at the sign-up stage would somewhat help to overcome this challenge and would provide key data on who participates and who is being reached (or not). An additional consideration for future realist evaluations is how best to craft a realist interview schedule for volunteers or service users who may have relatively low literacy levels or English as a second language. A key aim of a realist interview schedule is the testing of initial programme theories, which can often be quite complex or detailed in nature. Making these questions as accessible as possible to a range of participants needs to be balanced with the need to refine or refute the theories. Consequently, a plain English version of each theory can go some way to helping with any overly technical, specialist, or academic language.

### Strengths and limitations

A key strength of this research was the public and patient involvement and engagement through the entire lifecycle of the evaluation. The project had public members embedded on the team and advising at every stage, with local community members input and feedback on the research. Local community members were also involved as participants and then in an interactive dissemination and feedback workshop, which will help produce an accessible creative output about local experiences during COVID-19. The public and patient involvement and engagement throughout contributed to the range of practical recommendations and actions for local authority partners, which are linked to the programme theories (see Supplementary Table S2). A further strength is the use of a realist evaluation in the unique context of champion programmes during a pandemic, which allowed learning to be summarised across multiple programmes to inform future community centred approaches by local authorities.

This research has some inherent limitations, including the fact that despite the best efforts of the research and wider project teams, no true “service users” were involved as participants, because of the difficulty in identifying the programmes themselves. Therefore, we have limited data on “for whom” the programmes worked, a common feature of other localised evaluations of champion approaches which have not spoken directly to local “service users” [e.g., (9)]. There may be a key distinction between professional/trained and lay/volunteer community champion programmes, with the former often already aligned through their professional role with government and/or health related organisations [e.g., (5)]. They are, therefore, easily identifiable, and may be more willing to engage in an evaluation. The programmes evaluated in this study targeted groups that may have had distrust of the vaccine and associated organisations that were aligned to it. In addition, one downside of a realist evaluation is that it cannot provide strong evidence of whether the programmes affected key behavioural and health related outcomes such as vaccination or infection rates, or to what extent the programmes reached underserved groups. However, a recent evaluation of vaccine champion approaches in the UK found that some local authorities were reluctant to collect monitoring data as it was counter to informal and community-focused ethos of the programme and may undermine efforts to build trust (38). Even when data was collected there was no impact on vaccination uptake in residents of areas with Vaccine Champion funding (38).

## Conclusion

This study used a rigorous design and analysis approach to provide an original contribution to knowledge regarding the key implementation issues that can affect community champion and CPAR approaches during public health emergencies. Furthermore, a range of practical suggestions have been produced for local authorities, commissioners, and volunteer organisations to optimise programmes of this nature going forward. Representation and involvement of community members, establishing and building on existing trust, adequate and sustained training and resources, and clear communication, using a variety of delivery modes, from trusted community members and organisations are catalysts for meaningful engagement with communities. Local authorities should look to implement community champion and participatory research initiatives over the long-term so that a consistently proactive approach can be taken to improvement of services, while being better prepared to react to any future public health emergencies.

## Data availability statement

The original contributions presented in the study are included in the article/Supplementary material, further inquiries can be directed to the corresponding author.

## Ethics statement

The study involving humans were approved by the University of Hertfordshire Health, Science, Engineering & Technology Ethics Committee with Delegated Authority (ECDA): LMS/SF/UH/05067(2). The study were conducted in accordance with the local legislation and institutional requirements. The participants provided their written informed consent to participate in this study.

## Author contributions

NH: Conceptualization, Data curation, Formal analysis, Funding acquisition, Investigation, Methodology, Project administration, Supervision, Writing – original draft, Writing – review & editing. OF: Conceptualization, Data curation, Formal analysis, Investigation, Methodology, Project administration, Writing – review & editing. CB: Data curation, Formal analysis, Investigation, Project administration, Writing – review & editing. IS: Data curation, Formal analysis, Investigation, Writing – review & editing. LM: Conceptualization, Data curation, Formal analysis, Methodology, Writing – review & editing. AW: Conceptualization, Data curation, Formal analysis, Methodology, Supervision, Writing – review & editing. KB: Conceptualization, Data curation, Formal analysis, Funding acquisition, Methodology, Project administration, Supervision, Writing – review & editing.

## Footnotes

^1^At the time the programme ran in mid-2022 the UK living wage was £9.90 per hour. Source: https://www.livingwage.org.uk/what-real-living-wage

## References

[1] Jensen JA Bowers AP Gregson J. Transformations in Community Collaboration: Lessons from COVID-19 Champions across London (2023). Available at: https://www.adph.org.uk/networks/london/wp-content/uploads/sites/2/2023/02/Transformations_COVIDChampionsLondon_Feb2023_2.pdf (Accessed March 15, 2024).

[2] Public Health England. Community champions: a rapid scoping review of community champion approaches for the pandemic response and recovery. Public Health England (2021). Available at: https://www.gov.uk/government/publications/community-champion-approaches-rapid-scoping-review-of-evidence (Accessed March 15, 2024).

[3] Kok MC Dieleman M Taegtmeyer M Broerse JEW Kane SS Ormel H. Which intervention design factors influence performance of community health workers in low-and middle-income countries? A systematic review Health policy planning. (2015) 30:1207–27. doi: 10.1093/heapol/czu126, PMID: 25500559 PMC4597042

[4] Agarwal S Sripad P Johnson C Kirk K Bellows B Ana J. A conceptual framework for measuring community health workforce performance within primary health care systems. Hum Resour Health. (2019) 17:86. doi: 10.1186/s12960-019-0422-0, PMID: 31747947 PMC6868857

[5] Kaufman J Overmars I Leask J Seale H Chisholm M Hart J. Vaccine champions training program: empowering community leaders to advocate for COVID-19 vaccines. Vaccine. (2022) 10:1893. doi: 10.3390/vaccines10111893 PMC9693559 36366401

[6] Subramanian M Hemuka NJ Sivaramkrishnan D Chng NR Nightingale G Silva S. Evaluation of three west midlands local authority COVID-19 community champions programmes (2023). Available at: https://phirst.nihr.ac.uk/wp-content/uploads/2023/11/Research-Brief-CCC-PHIRST-Fusion-evaluation-17.11.23-1.pdf (Accessed March 15, 2024).

[7] Scientific Advisory Group for Emergencies. Role of Community Champions networks to increase engagement in context of COVID-19: evidence and best practice. (2020). Available at: https://www.gov.uk/government/publications/role-of-community-champions-networks-to-increase-engagement-in-context-of-covid-19-evidence-and-best-practice-22-october-2020 (Accessed March 15, 2024).

[8] South J Woodall J Stansfield J Mapplethorpe T Passey A Bagnall AM. A qualitative synthesis of practice-based learning from case studies on COVID community champion programmes in England. UK BMC Public Health. (2024) 24:24. doi: 10.1186/s12889-023-17470-1, PMID: 38166766 PMC10759547

[9] Newham.gov.uk. It takes a village (2022). Available at: https://www.newham.gov.uk/downloads/file/4255/newham-covid-19-champions-evaluation-report-feb-2022 (Accessed March 15, 2024).

[10] Dhaliwal BK Seth R Thankachen B Qaiyum Y Closser S Best T. Leading from the frontlines: community-oriented approaches for strengthening vaccine delivery and acceptance. BMC Proc. (2023) 17:5. doi: 10.1186/s12919-023-00259-w, PMID: 37391823 PMC10311705

[11] Micallef R Matharu R Barry A Burgess V. Description of a pharmacy COVID champion service in south East London to reduce vaccine hesitancy. Pharmacy. (2022) 10:143. doi: 10.3390/pharmacy10060143, PMID: 36412819 PMC9680247

[12] Ortiz K Nash J Shea L Oetzel J Garoutte J Sanchez-Youngman S. Partnerships, processes, and outcomes: a health equity–focused scoping meta-review of community-engaged scholarship. Annu Rev Public Health. (2020) 41:177–99. doi: 10.1146/annurev-publhealth-040119-094220, PMID: 31922931 PMC8095013

[13] Lindquist-Grantz R Abraczinskas M. Using youth participatory action research as a health intervention in community settings. Comm Health Promo. (2020) 21:573–81. doi: 10.1177/1524839918818831 PMC6692239 30577698

[14] George AS Mehra V Scott K Sriram V. Community participation in health systems research: a systematic review assessing the state of research, the nature of interventions involved and the features of engagement with communities. PLoS One. (2015) 10:e0141091. doi: 10.1371/journal.pone.0141091, PMID: 26496124 PMC4619861

[15] Brush BL Mentz G Jensen M Jacobs B Saylor KM Rowe Z. Success in long-standing community-based participatory research (CBPR) partnerships: a scoping literature review. Health Educ Behav. (2020) 47:556–68. doi: 10.1177/1090198119882989, PMID: 31619072 PMC7160011

[16] Breen LJ O’Connor M. From consultation to participation in public health research: reflections on a community-based research partnership. BMC Res Notes. (2014) 7:936. doi: 10.1186/1756-0500-7-936, PMID: 25527083 PMC4302106

[17] Marriott LK Lipus AC Choate L Smith J Coppola L Cameron WE. Experiential learning through participatory action research in public health supports community-based training of future health professionals. Pedagog Heal Promot. (2015) 1:220–32. doi: 10.1177/2373379915601119, PMID: 27536722 PMC4985246

[18] Brownson RC Shelton RC Geng EH Glasgow RE. Revisiting concepts of evidence in implementation science. Implement Sci. (2022) 17:26. doi: 10.1186/s13012-022-01201-y, PMID: 35413917 PMC9004065

[19] Dalkin S Greenhalgh J Jones D Cunningham B Lhussier M. What’s in a mechanism? Development of a key concept in realist evaluation. Implement Sci. (2015) 10:49. doi: 10.1186/s13012-015-0237-x, PMID: 25885787 PMC4408605

[20] Pawson R Tilley N. Realistic evaluation. London: Sage Publications Ltd (1997).

[21] Jagosh J MacAulay AC Pluye P Salsberg J Bush PL Henderson J. Uncovering the benefits of participatory research: implications of a realist review for health research and practice. Milbank Q. (2012) 90:311–46. doi: 10.1111/j.1468-0009.2012.00665.x, PMID: 22709390 PMC3460206

[22] Jagosh J Bush PL Salsberg J Macaulay AC Greenhalgh T Wong G. A realist evaluation of community-based participatory research: partnership synergy, trust building and related ripple effects. BMC Public Health. (2015) 15:1–11. doi: 10.1186/s12889-015-1949-1 26223523 PMC4520009

[23] Gov.uk. Vaccinations in United Kingdom (2022). Available at: https://coronavirus.data.gov.uk/details/vaccinations (Accessed September 28,20230.

[24] Dolby T Finning K Baker A Fowler-Dowd L Khunti K Razieh C. Monitoringsociodemographic inequality in COVID-19 vaccination uptake in England: a nationallinked data study. J Epidemiol Community Health. (2022) 76:646–52. doi: 10.1136/jech-2021-218415, PMID: 35470259

[25] Manzano A. The craft of interviewing in realist evaluation. Evaluation. (2016) 22:342–60. doi: 10.1177/1356389016638615

[26] Westhorp G Manzano A. Realist Evaluation Interviewing – A “Starter Set” ofQuestions (2017). Available at: https://www.ramesesproject.org/media/RAMESES_II_Realist_interviewing_starter_questions.pdf (Accessed March 15, 2024).

[27] Marchal B Dedzo M Kegels G. Turning around an ailing district hospital: a realistevaluation of strategic changes at ho municipal hospital (Ghana). BMC Public Health. (2010) 10:787. doi: 10.1186/1471-2458-10-787, PMID: 21184678 PMC3019197

[28] Bergeron DA Gaboury I. Challenges related to the analytical process in realistevaluation and latest developments on the use of NVivo from a realist perspective. Int J Soc Res Methodol. (2019) 23:355–65. doi: 10.1080/13645579.2019.1697167

[29] Gilmore B McAuliffe E Power J Vallières F. Data analysis and synthesis within a realist evaluation: toward more transparent methodological approaches. Int J Qual Methods. (2019) 18:160940691985975. doi: 10.1177/1609406919859754

[30] Razai MS Chaudhry UAR Doerholt K Bauld L Majeed A. Covid-19 vaccination hesitancy. BMJ. (2021) 373:n1138. doi: 10.1136/bmj.n1138 34016653

[31] Scottish Government. The impact of Covid-19 on communities, and priorities for recovery: Perspectives of organisations working in communities (2020). Available at: https://www.gov.scot/publications/impact-covid-19-communities-priorities-recovery-perspectives-organisations-working-communities/#:~:text=Participants%20in%20this%20research%20raised,of%20food%20and%20basic%20supplies (Accessed March 15, 2024).

[32] Haines A Sanders D Lehmann U Rowe AK Lawn JE Jan S. Achieving child survival goals: potential contribution of community health workers. Lancet. (2007) 369:2121–31. doi: 10.1016/S0140-6736(07)60325-0, PMID: 17586307

[33] Schleiff MJ Aitken I Alam MA Damtew ZA Perry HB. Community health workers at the dawn of a new era: 6. Recruitment, training, and continuing education. Heal Res Policy Syst. (2021) 19:113. doi: 10.1186/s12961-021-00757-3, PMID: 34641898 PMC8506097

[34] Gilmore B Ndejjo R Tchetchia A De Claro V Mago E Diallo AA. Community engagement for COVID-19 prevention and control: a rapid evidence synthesis. BMJ. (2020) 5:3188. doi: 10.1136/bmjgh-2020-003188 PMC7554411 33051285

[35] Mao G Fernandes-Jesus M Ntontis E Drury J. What have we learned about COVID-19 volunteering in the UK? A rapid review of the literature. BMC Public Health. (2021) 21:1470. doi: 10.1186/s12889-021-11390-8, PMID: 34320922 PMC8318044

[36] McCabe A Wilson M Macmillan R. Stronger than anyone thought: Communities responding to COVID-19. Local Trust (2020). Available at: https://localtrust.org.uk/insights/research/stronger-than-anyone-thought-communities-responding-to-covid-19/#:~:text=It%20identifies%20the%20varied%20responses,effective%20response%20to%20COVID%2D19 (Accessed March 15, 2024).

[37] Davies A Honeyman M Gann B. Addressing the digital inverse care law in the time of COVID-19: potential for digital technology to exacerbate or mitigate health inequalities. J Med Internet Res. (2021) 23:e21726. doi: 10.2196/21726, PMID: 33735096 PMC8030655

[38] IFF Research. Community vaccine champions evaluation report (2023). Available at: https://assets.publishing.service.gov.uk/government/uploads/system/uploads/attachment_data/file/1166982/Community_Vaccine_Champions_-_Evaluation_Report.pdf (Accessed March 15, 2024).

