# Supplementary material for: A realist evaluation of community champion and participatory action approaches during the COVID-19 pandemic
Source: Front Public Health. 2024 Jun 13;12:1355944. doi: 10.3389/fpubh.2024.1355944 (PMC11208485; doi:10.3389/fpubh.2024.1355944)
Supplement: Supplementary file 1 [file Table_1.docx]

| **Context** | **Mechanism resource** | **Mechanism reaction** | **Outcome** | **Decision** | **Final PT number (after refinement)** |
| --- | --- | --- | --- | --- | --- |
| C1a – Charity X has strong links into the Black African community in Southampton and has only recently started engaging with recently arrived refugee communities | Mres1 – Charity X uses existing networks and relationships with communities to promote champion/peer researcher opportunities | Mreact1 - Greater proportion of Black African community members than refugee community members feel that involvement in the programme is beneficial | O1 – More people from established Black African communities are recruited than from newly arrived refugee communities | Retain but with refinements | PT1 |
| C1b (rival theory) - Charity X has strong links into the Black African community in Southampton and has only recently started engaging with recently arrived refugee communities | Mres1a – Charity X goes out of its way to forge new relationships with the newly arrived refugee communities and uses them to promote champion/peer researcher opportunities | Mreact1b – Equal proportions of Black African and newly arrived refugee communities feel that involvement in the programme is beneficial | O1a – Equal proportions of people from the two communities are recruited | Not retained | n/a |
| C2a – If recruiting organisations have good existing knowledge of the communities they represent | Mres2a - Adverts will be tailored appropriately thus making the opportunity to be a champion/CPAR researcher more attractive | Mreact2a – Community members respond positively to the advertisement/message | O2a – They will achieve the greatest possible reach. | Not retained | n/a |
| C2b (rivalry theory) - If recruiting organizations do not have good knowledge or links with the communities they want to target | Mres2b – Adverts will be unrelatable to members in the community | Mreact2b – Community members do not respond positively to the advertisement and becoming a CPAR or champion is not viewed as ‘beneficial’ by those who see messaging and advertising | O2b – They will unlikely achieve a broad reach and recruitment will be unsuccessful. | Not retained | n/a |
| C3 – If recruited champions/peer researchers are a good representation of targeted communities | Mres3 – Champions/peer researchers will have more commonalities with the community | Mreact3 – This could lead to increased trust among members in the community who would perceive champions/peer researchers as more relatable | O3 – This can lead to increased engagement in the champion/peer researcher programme | Retained but refined into an alternative a and b version | PT2a and PT2b |
| C4 – If recruited champions/peer researchers are already well known in their communities/established leaders | Mres4 Champions/peer researchers will have existing links and networks with people across the target community | Mreact4 – Community members will be more receptive of champions/peer researchers as they would be perceived as trustworthy | O4 – This could lead to an uptake in the champion/peer researcher programme | Retained with refinements | PT3 |
| C5 – If Champions and CPAR researchers who might (or not) have prior relevant experience are adequately trained in the role | Mres5 - Key training materials and resources are provided | Mreact5 – Champions and CPAR researchers would feel more confident in their ability to deliver an effective service | O5 – This could lead to increased trust in the programme from community members | Retained with refinements | PT4 |
| C6 – If Champions and CPAR researchers have accurate expectations of their role | Mres6 – Provision of appropriate training and resources from organisations to volunteers, including expectations | Mreact6 – Champions and CPAR researchers feel that there is consistency between their expectations and the actual roles | O6 – Champions and CPAR researchers feel that their role is a positive experience and that they are helping their communities | Not retained | n/a |
| C7 – If champions/peer researchers perceive the importance of the programme | Mres7 – Champions/peer researchers are likely to be more committed in their role and establishing a good rapport with community members | Mreact7 – the commitment and enthusiasm of champions/peer researchers will mean community members pay attention to what they say | O7 – This will likely facilitate reach, engagement, trust, understanding and strengthen community engagement in the programme | Retained with refinements | PT5 |
| C8 – If there is limited prior relationship between champions/peer researchers and the target communities | Mres8 – Time, patience and consistency will need to be invested | Mreact8 – Community members will be more receptive towards the programme | O8 – This can lead to meaningful reciprocal engagement between champions/peer researchers and community members | Retained but combined with PT2a and 2b | PT2a and PT2b |
| C9 – If there is a sufficient amount of representative community members engaging in the programmes | Mres9 – This provides a forum for local authority and partner organisations and community members to understand each other | Mreact9 – Local authority and partner organisations would feel more confident in their understanding about how to meet the needs of community members and community members would feel more trusting of services | O9 – This improves perceptions and relationships between local authority and partner organisations and community members | Retained with refinements | PT6 |
| C10 – Insights from community members about how best to deliver current programmes are gained from champions/peer researchers | Mres10 – Changes in the time/location/context/delivery mode of current programmes are made | Mreact10 – Community members feel heard and listened to when changes to programmes are made | O10 – Programme uptake/engagement is improved among target communities/groups/individuals | Retained but refined into an alternative a and b version | PT7a and PT7b |
| C11 – Insights from community members about how best to deliver future services are gained from champions/peer researchers | Mres11 – Changes in the time/location/context/delivery mode/content of future services are made | Mreact11 – Community members feel heard and listened to when changes to services are made | O11 – Service uptake (and wider health and wellbeing outcomes) is improved among target communities/groups/individuals | Retained but combined with PT7a and 7b | PT7a and PT7b |
| C12 – If recruited champions/peer researchers have a good understanding of the importance of the programme | Mres12 – Champions/CPAR researchers are likely to be more empathetic and sensitive in their approach to engagement with community members and council/services | Mreact12 – Champions/CPAR researchers are likely to feel more passionate and committed to their role | O12 – Better engagement could be facilitated between community members and council/services and Champions/CPAR researchers in the programme | Retained but combined with PT5 | PT5 |
| C13 – If recruited champions/CPAR researchers have personal experience of the health and wellbeing experiences or circumstances of the target individuals | Mres13 – Champions/CPAR researchers will have better knowledge and more understanding | Mreact13 – This could lead to increased trust amongst targeted individuals | O13 – This could lead to increased and more meaningful engagement with the champion/peer researcher programmes | Retained with refinements | PT8 |
| C14 – If there is a supportive infrastructure in place and long-term commitment to community engagement | Mres14 – The provision of greater social connections and better linking of communities and services | Mreact14 – Champions/CPAR researchers would feel more knowledgeable, a sense of autonomy, and what they are doing is making a difference | O14 – Greater reach would be facilitated by Champions/CPAR programmes into underserved communities, to inform future projects and services | Not retained | n/a |
| C15 – If there is a lack of sustainable resources for volunteer champions and partner organisations | Mres15 – Champions will be less enabled to perform the duties of the role and be stretched when they do | Mreact15 – Existing volunteer champions and partner organisations are likely to feel stressed and burnout, and might lose trust in the approach | O15 – The longevity of existing volunteers will be threatened, and Champion programmes are in danger of not achieving ongoing reach into and collaboration with underserved communities | Retained with refinements | PT9 |
| C16 - If there is a lack of accessible physical locations to get vaccinated | Mres16 - Champions are not supported by accessible services to engage the community | Mreact16 - Champions feel less agency to perform their role well | O16 – This could result in a lack of trust and confidence between the community and champions, and subsequently the service providers | Retained with refinements | PT10 |
| C17 - If there is no appropriate service to meet the needs of the community (e.g., language, disability, layout of service model such as single sex option) | Mres17 - Champions are not supported by accessible services to engage the community | Mreact17 - Champions feel less agency to perform their role well | O17 – This could result in a lack of trust and confidence between the community and champions, and subsequently the service providers | Retained with refinements | PT22 |
| C18 – If individuals within the community have a lack of trust in pharmaceutical companies and government organisations such as NHS/council | Mres18 – Conversations with champions can address the beliefs held which lead to the fear/lack of trust and reassure individuals within the community | Mreact18 – Individuals within the community are less likely to feel sceptical about the vaccine and to feel more comfortable with the Champion programmes | O18 – This would lead to changed attitudes about the vaccine in community members | Retained with refinements | PT11 |
| C19 – If individuals in the community do not feel as though they have enough information about the contents of the vaccine or the possible side-effects | Mres19 – Communication with champions will educate and provide the accurate information | Mreact19 - Individuals trust the Champions and feel well-informed about vaccine contents/side effects | O19 – This would lead to better understanding of the vaccine and stronger intentions to get the vaccine | Retained with refinements | PT12 |
| C20 – If individuals in the community are complacent about their risk of contracting COVID-19 and the potential impact on their lives (including loved ones) | Mres20 – Champions can provide accurate information and resources to individuals within the community | Mreact20 – individuals/community members would assimilate new information | O20 - This would lead to changed perceptions (i.e. less complacency) about the necessity of self-protective behaviours such as physical distancing and mask wearing | Retained with refinements | PT13 |
| C21 – If individuals in the community have had bad experiences of vaccines in the past | Mres21 – Champions can provide support via one-to-one interactions or group engagements | Mreact21 – Individuals are likely to feel more reassured and well-supported | O21 – This can lead to changed perceptions and attitudes towards the programme and subsequently lead to increased engagement | Retained with refinements | PT14 |
| C22 – If information about the vaccine is not readily accessible to individuals in the community | Mres22 – Champions can act as the bridge in providing key information to individuals within the community | Mreact22 – Individuals in the community would feel that they have better understanding and knowledge of the vaccine and related issues | O22 – This would likely lead to more positive attitudes towards vaccination | Retained with refinements | PT15 |
| C23 – If Vaccine Champion organisations already represent community members and/or issues of importance (e.g., health, faith) | Mres23 – They are ideally positioned as trusted sources to circulate key information | Mreact23 – community members are more likely to pay attention to messages they hear from trusted sources | O23 – Engagement and impact is more likely with community members | Retained with refinements | PT16 |
| C24 – If individuals in the community do not feel empowered to make their own decisions in respect to taking the vaccine | Mres24 – Interactions with CPAR researchers can give individuals opportunities to explain their experiences and concerns | Mreact24 | O24 – Individuals in the community are less likely to feel ignored or disenfranchised | Retained with refinements | PT17 |
| C25 – If appropriate support structures are in place for the CPAR programme to engage with local organisations | Mres25 – The CPAR programme provides a tangible way for public health programmes to work closely with community organisations | Mreact25 - | O25 – This can lead to effective ongoing partnerships with community organisations | Retained with original wording | PT18 |
| C26 - For CPAR researchers that would like to be involved in community-related activities or health-related careers in the future | Mres26 - Well-designed training and sustained resources to support community engagement | Mreact26 - CPAR researchers perceive the resources as valuable learning and experience for future fields of interest (e.g., future nursing and medicine-related careers) | O26 – This can lead to better experience and preparation for future employment | Retained with refinements | PT19 |
| C27a – If there is a lack of sustainable resource for the CPAR approach | Mres27a - Lack of effective training and information for community researchers | Mreact27a – CPAR researchers are likely to feel disinterested in participating in the programme and less committed | O27a - CPAR programme may not achieve good relationships with underserved communities | Retained with refinements | PT17 |
| C27b – If there is sustainable resource for the CPAR approach | Mres27b - Effective training and information exchange for community researchers | Mreact27b – CPAR researchers are likely to feel interested in participating in the programme and committed | O27b – The CPAR programme is more likely to achieve good relationships and collaboration with underserved communities | Retained with refinements | PT20a and PT20b |
| C28 – Local organisations may have a lack of expertise and capacity for community research | Mres28 – The CPAR programme brings training and support for organisations to hear from local residents in a structured way | Mreact28 | O28 - More capacity in the system for community organisations to find out the needs and experiences of their communities and to cascade this knowledge to other people in the organisations | Retained with original wording | PT21 |
