# Supplementary material for: A realist evaluation of community champion and participatory action approaches during the COVID-19 pandemic
Source: Front Public Health. 2024 Jun 13;12:1355944. doi: 10.3389/fpubh.2024.1355944 (PMC11208485; doi:10.3389/fpubh.2024.1355944)
Supplement: Supplementary file 2 [file Table_2.docx]

**Supplementary Table 2 – Final programme theories, plain English summaries, and take-home messages**

| Programme theory number and label | Final programme theory | Plain English Summary | Take home messages |
| --- | --- | --- | --- |
| 1 - Reaching potential champions and peer researchers | *Context* – If community organisations have strong links into target groups such as the Black African community.  *Mechanism Resource* – Community organisations can use existing networks and relationships with communities to promote COVID-19 champion/peer researcher opportunities.  *Mechanism Reaction* – Greater trust and knowledge of perceived barriers and cultural beliefs are gained.  *Outcome* – More people from target groups are recruited as COVID-19 champions/peer researchers | For recruitment of COVID-19 champions and peer researchers, local community organisations are well placed, with good existing networks to promote the roles, particularly to people from relevant groups (e.g., the Black African community). Because of their knowledge of potential barriers and cultural issues, there is greater trust and, therefore, more people from target groups will take up the roles. | - Recruitment through existing local community organisations is ideal - Recruiting online or through other avenues unconnected to these groups is less likely to produce representative champions and peer researchers |
|  |  |  |  |
| 2 - Getting representative champions and peer researchers | *Context* – If recruited champions/peer researchers are a good representation of targeted communities  *Mechanism Resource* – Champions/peer researchers will have more commonalities with the community and will be more likely to have the confidence, knowledge, and understanding to approach the communities.  *Mechanism Reaction* – This could lead to increased trust among members in the community who would perceive champions/peer researchers as more relatable  *Outcome* – This can lead to increased engagement from community members in the champion/peer researcher programme  Alternative  *Context* – If recruited champions/peer researchers are a good representation of targeted communities  *Mechanism Resource* – Champions/peer researchers will have more commonalities with the community and will be more likely to have the confidence, knowledge, and understanding to approach the communities.  *Mechanism Reaction* – This could lead to a reluctance among members in the community to share their views with the champions/peer researchers because they belong to a tight-knit community who know each other, and they fear what they say may become known by others in that community  *Outcome* – This can lead to decreased engagement from community members in the champion/peer researcher programme | Being peers of community members, integrated into the community, and/or looking and sounding like other community members was viewed in a mixed way in terms of the possible effect on trust and engagement from others.  Some people viewed it positively because of the shared experiences, similarities, and history between researchers/champions and community. Others viewed it negatively as it might lead to community members not wanting to be as open and honest because of fears that these views would get back to other community members. | - The team agreed that this theory was largely focused on the CPAR programme - The representativeness of peer researchers could result in getting a lot of insight from community members that might not have been possible without this connection - This is weighed up against the view that the opposite could also happen where the shared history or culture could lead people to not want to divulge detailed information about their thoughts and feelings - Future programmes need to weigh these pros and cons when recruiting peer researchers. It may depend on the topic(s) under investigation as to the appropriate approach. |
|  |  |  |  |
| 3 - Embedded champions and peer researchers | *Context* – If recruited champions/peer researchers are already well known in their communities/established leaders  *Mechanism Resource* - Champions/peer researchers will have existing links and networks with people across the target community  *Mechanism Reaction* – Community members will feel safe and be more receptive of champions/peer researchers as they would be perceived as trustworthy  *Outcome* – This could lead to increased uptake and meaningful reciprocal engagement between champions/peer researchers and community members, which can last over the long-term  Alternative  *Context* – If recruited champions/peer researchers are not already well known in their communities/established leaders  *Mechanism Resource* - Champions/peer researchers will have to spend time attending and establishing themselves within community groups  *Mechanism Reaction* – Community members will feel safe and be more receptive of champions/peer researchers as they would be perceived as trustworthy  *Outcome* – This could lead to increased uptake and meaningful reciprocal engagement between champions/peer researchers and community members, which can last over the long-term | It is important to recruit champions or peer researchers that are either well known and/or community leaders because they are linked into their communities and will be seen as safe and trustworthy.  This will mean members of the public are more receptive to them and they will have more meaningful discussions with each other | - Familiarity with champions and peer researchers is an important facilitator of programme success. This was particularly important for peer researchers who were asking for people’s in-depth thoughts and feelings in interviews - This familiarity can be achieved through both being an established leader or being known to community members - Being known can be achieved, even for relatively new people, if they embed themselves in community groups before trying to engage in programme-related activities - There was a notion that if community members perceived the champions or peer researchers as strangers then having a way to verify who they were was important - Reciprocal engagement was seen as both the ability to provide accurate information (often quickly) to community members and to get clear information on their needs - There were also comments about the benefits to the mental health of champions, in that they felt that were making a difference to people’s lives during tough times |
|  |  |  |  |
| 4 - Enabling champions and peer researchers | *Context* – If champions/peer researchers who might (or not) have prior relevant experience are adequately trained in the role  *Mechanism Resource* - Timely training materials and resources are provided, with opportunities to practice and access to ongoing support or mentoring  *Mechanism Reaction* – Champions/peer researchers would feel more confident and enabled to deliver an effective service  *Outcome* – This could lead to more effective reach into communities and communications with community members | For champions/peer researchers, regardless of their experience level, training materials and resources that are provided at an appropriate time (i.e., close to when they have to go out into the field) are important. When combined with further opportunities to practice these skills, this will make them feel more confident in the role and allow them to have successful conversations with community members. | - Champions/peer researchers can feel confident in their role, even if they have not got prior experience, with timely training - Further support and practice helps confidence levels and embeds skills from the training - More confident and well-practiced champions/peer researchers can have effective communications with community members |
|  |  |  |  |
| 5 - Commitment facilitates reach | *Context* – If COVID-19 champions and/or peer researchers perceive the importance of the programme  *Mechanism Resource* – COVID-19 champions and/or peer researchers are likely to be more committed to helping the local communities with their health and related needs  *Mechanism Reaction* – The commitment and enthusiasm of COVID-19 champions and/or peer researchers will mean community members see them as more invested  *Outcome* – This could facilitate reach, engagement, trust, understanding and strengthen community engagement in the programme | Individuals who signed up to be a COVID-19 champion or a peer researcher often saw their involvement as important in helping their community. The commitment and enthusiasm that they brought to the role, drove them to reach and engage with community members, who were likely to see them as invested in understanding and helping them. | - This programme theory was more representative of COVID-19 champions and peer researchers than the typically more organisation level vaccine champions - It helps programmes of this type when they recruit individuals who are invested in helping their community and see the role as important in supporting community members - It may have been easier for people to feel this role was important because of the nature of the covid-19 pandemic context – we were in a crisis situation. It may be harder to convince people of the importance of this type of role outside of a crisis situation. - There was evidence that taking on this role brought benefits to the mental health of champions, in that they felt that were making a difference to people’s lives during tough times |
|  |  |  |  |
| 6 - Two-way understanding and ownership | *Context* – If there are representative community members engaging in the programmes  *Mechanism Resource* – This provides a forum for local authority and partner organisations and community members to listen to and understand each other, through champions  *Mechanism Reaction* – Local authority and partner organisations would feel more confident understanding how to meet community member needs and community members would feel more empowered, and trusting of services  *Outcome* – This improves relationships between local authority and partner organisations and community members, and brings about a sense of shared ownership of programmes/services | It is important that champions engage with community members from groups that are underserved in the community. This allows community members, champions, and organisations to be able to talk to and better understand each other. Community members would then feel more trusting of services and feel empowered by a sense of shared ownership of future programmes or services. | - Reaching and engaging meaningfully with community members that represent underserved communities is important - Providing a forum for two-way engagement between these community members and the local council/organisations will improve relationships. The champions are the catalysts for this by passing on important information in both directions - Providing a way for community members to collaborate on the approach that future programmes and services might take will increase buy in and engagement going forward - There were comments to suggest that people in the community, and as part of groups, were acting as informal champions (without having signed up) in guiding and helping others in support networks |
|  |  |  |  |
| 7 - Insights must lead to improvement of services | *Context* – If insights from community members about how best to deliver current programmes, and potential barriers are gained through champions/peer researchers  *Mechanism Resource* – Changes to the current programmes are made (e.g., time/location/activities)  *Mechanism Reaction* – Community members feel heard and listened to when changes to programmes are made  *Outcome* – Programme uptake/engagement is improved among target communities/groups/individuals  Alternative  *Context* – If insights from community members about how best to deliver current programmes, and potential barriers are gained through champions/peer researchers  *Mechanism Resource* – There are no actions taken in terms of changes to the current programmes or future services  *Mechanism Reaction* – Community members feel disappointed by the lack of actions/improvements  *Outcome* – Uptake/engagement is negatively affected for current programmes and future services among target communities/groups/individuals | Discussions with community members bring important understanding about how to deliver appropriate services and the barriers there might be for them to engage.  Then either visible changes are made, or they are not (or very limited changes are made). Community members then either feel like services have responded to their views or they feel ignored.  Depending on the reaction of the services, the level of involvement in current and future programmes from target groups can be really different. | - If programmes focus their ethos on gaining insights from community members and embracing co-creation/design, it is really important they do this in a meaningful and transparent way so that community members can see changes as a result - There is a real danger in alienating community members if they feel like programmes are paying ‘lip-service’ to consulting them, but then nothing changes - This theory and learning are particularly relevant for the CPAR programme where people interviewed often felt like there had not been enough or any action |
|  |  |  |  |
| 8 - Shared experiences support engagement | *Context* – If recruited Champions/peer researchers have personal experience of the health and wellbeing experiences or wider circumstances of the target individuals  *Mechanism Resource* – Champions/peer researchers will have better understanding and be more able to relate  *Mechanism Reaction* – Target individuals feel better understood and more able to trust champions/peer researchers  *Outcome* – This could lead to increased and more meaningful engagement with the champion/peer researcher programmes | Having personal experience of the circumstances of community members (e.g., health condition, housing, deprivation) allows the champions/peer researchers to understand and relate more, leading to increased trust from community members, which is more likely to lead to meaningful engagement from them. | - Personal experience of or familiarity with the circumstances can be wider than health or wellbeing issues and can be relevant for any challenges being experienced - There were also comments suggesting group identification could be another way to be familiar and trustworthy (e.g., being a veteran) - It was also stated that good training can somewhat make up for this lack of personal experience, by helping peer researchers to develop the necessary skills and knowledge. Moreover, training allows champions/peer researchers to ask questions in the right way to elicit views/comments about these issues during interviews |
|  |  |  |  |
| 9 - Sustainable resources help reach and impact | *Context* – If there is a lack of appropriate and sustainable resources (e.g., funding, capacity) for volunteer champions and partner organisations (vaccine and CPAR)  *Mechanism Resource* – Champions and partner organisations will be less enabled to perform the duties of the role and be stretched when they do  *Mechanism Reaction* – Existing volunteer champions and partner organisations are less likely to engage or continue to engage with the programme  *Outcome* – The longevity of existing volunteer champions and partner organisations will be threatened, and they are in danger of not achieving ongoing reach into and collaboration with underserved communities (where some vulnerable communities might require greater resource). | Sustainability of resources for both individuals and partner organisations are key to helping champions and community researchers to perform their role, and to continue to be part of the programmes. This is particularly important when trying work with communities that might be vulnerable and need more help. | - Some communities can be engaged with in a fairly light touch way, but some vulnerable communities require greater resource. - Zoom, email, and internet connection were mentioned as useful resources for champions to ensure fast communication. - Peer researchers mentioned things like having a laptop or childcare as important resources to help their role. - The original wording mentioned stress and burnout as possible reactions to lack of sustainable resources, but the feedback was much more related to engagement levels dropping. For example, some volunteers would like to continue but cannot without funding |
|  |  |  |  |
| 10 - Good availability of services supports champions | *Context* - If there are a range of accessible physical locations to get vaccinated  *Mechanism Resource* - Champions are supported by accessible services to engage the community  *Mechanism Reaction* –  *Outcome* – Champions feel more agency to perform their role well | Having a range of places that people can access for vaccinations played an important role in supporting Champions to encourage vaccinations. Without this it would have been a much more difficult job | - Originally this theory was negatively framed about the dangers of a lack of locations. The comments from interviews showed that the general view was there were ample locations for vaccinations at the time. - The location and availability of vaccinations centres was deemed important, but also the amount of people around was also mentioned, due to worries about COVID transmission. Cost of parking and petrol for long distances were also concerns. - Being familiar with the space and the people was a facilitator in receiving a vaccine in community hubs (e.g. mosque). - One comment mentioned that without accessible vaccine centres there is more resistance to vaccine uptake, but this was not enough to change the outcome overall |
|  |  |  |  |
| 11 - Changing attitudes when trust is low | *Context* – If individuals within the community have a lack of trust in pharmaceutical companies and government organisations such as NHS/council  *Mechanism Resource* – Conversations with champions about COVID-19 generally and the vaccine can give the necessary and accurate information via informal conversation or online communication which can help alleviate concerns and reassure individuals in the community.  *Mechanism Reaction* – Individuals within the community are less likely to feel sceptical / suspicious about the vaccine and to feel more comfortable with the Champion programmes.  *Outcome* – This would lead to changed attitudes about the vaccine in community members. | It was felt that people could have a lack of trust in pharmaceutical companies and government organisations. When this was the case, champions could provide accurate information, which might help with concerns and reassurance. These chats/communications could lead to less scepticism and more positive attitudes about the vaccine and COVID-19 more generally. | - There was a distinction drawn between pharmaceutical companies and government organisations/NHS. The latter was viewed as more trustworthy as an ‘authority’. - There was a feeling that it is not the role of the champions to change the community members mind about trusting the pharmaceutical company, but only to give them information that would provide reassurance (i.e., they would be fine if they had the vaccine) - Embedding the communications within the normal practice of vaccine champion organisations was viewed as best practice, so it wasn’t adding a whole new workstream but making it part of business as usual - One comment mentioned that this information provision might have led to greater vaccine uptake, but this was not enough to change the outcome wording overall. |
|  |  |  |  |
| 12 - Personable and appropriate communication | *Context* – If individuals in the community do not feel as though they have enough vaccination information, or the information keeps changing  *Mechanism Resource* – Communication with champions and peer-researchers provide personable, accurate and up-to-date information without pressure, or sign-posting to a suitable health professional who can provide specific information about vaccine side-effects  *Mechanism Reaction* - Individuals trust the Champions and peer-researchers and feel well-informed  *Outcome* – This can lead to better understanding of the vaccine and stronger intentions to get the vaccine | Community members can feel they haven’t got enough information, or that the advice keeps changing. Champions and/or peer researchers can provide accurate information in a personal way, and when they have not got the expertise, they can refer the community member onto someone who has. Community members then trust the champions and/or peer researchers, feel well-informed, and might be more likely to get the vaccine. | - There were comments about the trustworthiness of information around the vaccine and the changing information (e.g., it turns out the vaccine did not prevent virus transmission very well). In a fast-paced evolving situation honesty about what is not yet known is therefore important - It was generally thought that the champions/peer researcher should not be talking about vaccine contents and side-effects, which was more suitable for a health professional - It was, however, believed that information, even if it was fairly basic, was more effectively communicated by champions because it was personal and more story like (rather than stats and graphs). - There was importance placed on the method of communication the Champion employs and how approachable they are. Applying pressure to the individual could put them off, the recipient may prefer to receive the information and then be left to make their own decision. |
|  |  |  |  |
| 13 - Communicating risk to combat complacency | *Context* – If individuals in the community are complacent (or do not agree) about their risk of contracting COVID-19 and the potential impact on their lives (including loved ones)  *Mechanism Resource* – Champions can provide accurate information about risk to individuals in the community, and their loved ones  *Mechanism Reaction* –  *Outcome* - This would lead to individuals making more informed choices and increased awareness about the needs of others | Individuals in the community may not be worried or hold different opinions regarding their risk of getting COVID-19 and the impact it might have on their lives and loved ones. Champions can provide accurate information about the risk of COVID-19 to individuals in the community and educate them about the potential impact on their loved ones.    By sharing this information, the champions can help community members to make more informed choices and increase their awareness of the needs and well-being of others. The champions' responsibility is to provide accurate information regardless of whether community members are willing to listen. This ensures that community members are well-informed before making decisions related to protecting themselves and others. | - There was a feeling that some people will not change their minds and that all you can do is provide information - The outcome was more about giving people the ability to have an informed choice rather than changing perceptions - There was however a sense that providing information could increase awareness of others' needs/vulnerability. |
|  |  |  |  |
| 14 - Addressing negative vaccine experiences | *Context* – If individuals in the community have had bad experiences of vaccines in the past  *Mechanism Resource* – Champions can provide support via one-to-one or group interactions  *Mechanism Reaction* – Individuals are likely to feel more reassured and well-supported, as long as a group setting does not bring about negative peer influence  *Outcome* – This can lead to changed attitudes towards the vaccine, and potentially greater likelihood of taking it again in future | Some community members have had negative experiences with vaccines in the past. Champions can provide support by listening to the concerns of community members.    By talking with champions, individuals are more likely to feel reassured and well-supported throughout the process. However, it is important to consider the potential negative influence of strong opinions within a group setting, as it may sway individuals against the vaccine. Nonetheless, the support and guidance offered by champions can help shift attitudes among community members. This change may result in an increased chance of individuals considering and even accepting the vaccine in the future. | - Comments highlighted experiences like sore arms/bruising, feeling ill, and it not protecting people against infection (all COVID vaccine related). - There were potential downsides highlighted related to group discussions, such as feeling less comfortable to share concerns in a group, the effect of peer pressure, or the impact of the rest of the groups opinions. - Making use of normal interactions with patients or members of the public so that the conversation is part of business as usual for staff and organisations is likely to be helpful. - Programme engagement and attitudes were not mentioned. From the comments, a change in confidence and/or better chance of vaccine uptake in future was more likely. |
|  |  |  |  |
| 15 - Making informed choices | *Context* – If information about the vaccine is not readily accessible to individuals in the community.  *Mechanism Resource* – Champions can act as the bridge in providing key information to individuals within the community and feedback the community voice.  *Mechanism Reaction* – Individuals in the community would feel that they have better understanding and knowledge of the vaccine and related local and country wide issues.  *Outcome* – This would likely lead to individuals being able to make a more informed choice about vaccination and the associated community benefits. | Champions can be more approachable and share information about vaccines directly to their local communities. Additionally, any concerns raised by community members can be fed back directly to community organisations and/or the public health team. Champions who live within communities can share the most relevant vaccine knowledge, especially when it comes to specific local issues, as well as wider national debate. This will help people make informed decisions on vaccination, and the associated benefits for themselves, and the local community. | - Direct contact with the local communities meant specific concerns could be addressed e.g., where to go and how to book your vaccine. - Increasing vaccine knowledge led to people being able to make a more informed decision. People felt they had more autonomy in their decision - ‘take things into their own hands’. - Through gaining a better understanding of vaccine related community benefits – people can see how their actions may be affecting the bigger picture. - Champions can act as a community voice, with local understanding, to either provide or advocate for relevant vaccine information for people with a range of needs. This can create a ‘tangible connection’ through increased trust between community members and organisations. |
|  |  |  |  |
| 16 - Representation through vaccine champion organisations | *Context* – If Vaccine Champion organisations already represent local community members and/or issues of importance (e.g., health, faith)   *Mechanism Resource* – They are ideally positioned as trusted sources to initiate conversation and circulate key information   *Mechanism Reaction* – Community members are more likely to pay attention to messages they hear from trusted sources, especially where direct contact has been made.  *Outcome* – A trusted source is gained in the champions, who are actively involved in local matters. This can lead to community members making more informed decisions, thereby increasing engagement and impact. | Vaccine champions who represent and are established within local communities will be more trusted when it comes to circulating vaccine information. Direct conversation with community members helps people trust the information that is being shared and can help people make informed decisions. Sharing knowledge within communities can increase the engagement in and impact of vaccine programmes. | - Direct contact and engaging communication, through established organisations created a safe and trusted source (e.g., one interviewee’s opinion on vaccination changed through conversations at their mosque.). - Vaccine information could be shared though established community links, but questions could also be referred back to community organisations and/or the public health team. - Champions act as a community ‘beacon’ getting information directly and increasing community engagement. - It was also commented that the champions enhanced community ‘pride’ and ‘reputation’. |
|  |  |  |  |
| 17 - Feeling heard and informed | *Context* – If individuals in the community do not feel empowered or informed enough to make decisions in respect to taking the vaccine  *Mechanism Resource* – Interactions with CPAR researchers can give an opportunity to either explain experiences and concerns, get accurate information, and/or be referred to someone more knowledgeable  *Mechanism Reaction*  *Outcome* – Individuals in the community are less likely to feel ignored or misinformed | Community members can feel like they are not in control of their own decisions about whether to take the vaccine or not, and that they make lack information, or a way of explaining their concerns. Speaking with a CPAR researcher could allow them to explain their concerns and be directed towards someone with medical training for more technical questions. This would meant that community members would feel they have had their voice heard. | - It was not considered the role of the CPAR researchers to persuade or convince the community member of anything. - Often the need to speak to people with greater expertise (e.g., a GP) was mentioned as important - The importance of feeling heard and the opportunity of having a two-way dialogue was consistently mentioned - Empowerment and personal choice were seen as a key factor for community members |
|  |  |  |  |
| 18 - Partnership working improved | *Context* – If appropriate support structures are in place for the CPAR programme to engage with local organisations  *Mechanism Resource* – The CPAR programme provides a tangible way for public health programmes to work closely with community organisations  *Mechanism Reaction*  *Outcome* – This can lead to effective collaboration and ongoing partnerships with community organisations | The CPAR programme provided a way for some good partnership working between public health, voluntary services, and community organisations. This has provided a useful blueprint for working together in the future | - A number of people stated that the CPAR programme was beneficial for different organisations to work together - There was agreement that this provided a vision for future partnership working going forward to help tackle a range of issues |
|  |  |  |  |
| 19 - Experience for future career paths | *Context* - If CPAR researchers would like to be involved in community-related activities or health-related careers in the future  *Mechanism Resource* - Well-designed training and sustained resources to support community engagement  *Mechanism Reaction* - CPAR researchers perceive the resources as valuable learning and experience for future careers (e.g., healthcare-related), even if they had not thought about this before  *Outcome* – This can lead to a better experience and preparation for future employment, and the broadening of horizons for those new to community research | The CPAR programme, involved strong training and resources for support, which were seen as valuable for people planning a career in community/health related areas. Even when there were no initial plans for a career, it was seen as a great opportunity for those that would not have a chance otherwise to access this type of training | - The initial framing of this programme theory was around people who were purposefully building up skills and experience for their CV. Comments suggested that there were also people who may never have considered this avenue before this opportunity arose. This suggests it could be a means for social mobility if offered to a range of people - The accredited qualification that was on offer after the CPAR programme had finished was considered a really valuable element of involvement as a peer researcher |
|  |  |  |  |
| 20 - Resources and training for community research | *Context* – If there is a lack of adequate resource for the CPAR approach  *Mechanism Resource* - Lack of effective training and ongoing support for community researchers. CPAR research might not be conducted in a safe, ethical, confidential, or open-ended way  *Mechanism Reaction* –  *Outcome* – The CPAR programme is not viewed as trustworthy and community members do not feel comfortable being open and honest in interviews  Alternative  *Context* – If there is adequate resource for the CPAR approach  *Mechanism Resource* - Effective training and ongoing support for community researchers.  *Mechanism Reaction -* CPAR researchers are able to conduct safe, ethical, confidential, and open-ended interviews.  *Outcome* – The CPAR programme is viewed as trustworthy and community members feel comfortable being open and honest in interviews, providing valuable insights | Appropriate resources and training for the peer researchers was seen as essential to gain the trust of the community members interviewed, which enabled them to be honest and provide their views. The danger of not having the right resources or training was the potential for peer researchers to conduct interviews in a leading way, not fully respecting confidentiality, and not behaving in an ethical manner, which could jeopardise the trust and safety of community members | - The quotes suggested that sustainable resourcing and training was seen as essential for peer researchers. - However, our original wording focused on being disinterested, but this was clearly not the main concern for researchers if adequate training had not been given - Greater concerns and follow-on outcomes were: Safeguarding of researchers and community members; Not being able to handle (unequipped) difficult conversations; Being mindful not to ask leading questions and dictate interviews; Maintaining confidentiality and being trustworthy; Community members would not feel comfortable being open and honest; maintaining ethical standards. |
|  |  |  |  |
| 21 - Community capacity building achieved | *Context* – If local organisations have a lack of expertise and capacity for community research  *Mechanism Resource* – The CPAR programme brings training and support for organisations to hear from local residents in a structured way  *Mechanism Reaction*  *Outcome* - More capacity in the system for community organisations to find out the needs and experiences of their communities and how to effectively meet these. Moreover, this knowledge can be cascaded to other people in the organisations | Local organisations may have had the desire to do community research but haven’t had the capacity and/or expertise. The CPAR programme provided this capacity to get to the heart of the issues that were important to community members, with the possibility to pass this knowledge onto other colleagues | - Training community members and/or organisation volunteers to be peer researchers is a good way to generate additional capacity in local organisations - This additional capacity can then be utilised in future and passed down through organisations (with appropriate support and guidance) |
|  |  |  |  |
| 22 - Champions supported by accessible services | *Context* - If there is no appropriate service to meet the needs of the community (e.g., language, disability, layout of service model such as single sex option) or Champions do not have the resources to meet the need of the job (e.g. a quiet place to talk)  *Mechanism resource* - Champions are not supported by accessible services and resources to engage the community  *Mechanism react* - Champions feel less agency to perform their role well and community members may feel the Champion should be able to find alternatives that meet the need  *Outcome* – This could result in a lack of trust and confidence between the community and champions, and subsequently the service providers | The community’s trust and confidence in Champions may be jeopardised if Champions are not supported by availability of required resources (e.g. a private place to chat) to do the job, or an appropriate service to carry out the vaccination (e.g. an accessible site). The effect on Champions may include feeling less able to perform their role well. | - Agreement from community members constituted that championing something that there isn’t the resource or service for could undermine trust and confidence in Champions. - However, some members of the public felt it was the responsibility of the Champion to find alternatives and direct the member to a service that can meet their need (e.g. an accessible vaccination centre). |
